# Clustering of self-reported social and environmental risk factors identifies vulnerable subgroups with increased asthma morbidity

**DOI:** 10.1016/j.jacig.2026.100775

**Published:** 2026-08-14

**Authors:** Alana Schreibman, Kimberly Lactaoen, Anuradha L. Vyas, Lizbeth F. Gómez, Aurora Mills, Patrick K. Gleeson, Andrea J. Apter, Rebecca A. Hubbard, Gary E. Weissman, Blanca E. Himes

**Affiliations:** a Harvard Medical School, Boston, Mass; b Department of Biostatistics, Epidemiology and Informatics, Perelman School of Medicine, University of Pennsylvania, Philadelphia, Pa; c Division of Pulmonary, Allergy and Critical Care Medicine, Perelman School of Medicine, University of Pennsylvania, Philadelphia, Pa; d Department of Biostatistics, School of Public Health, Brown University, Providence, RI; e National Heart, Lung, and Blood Institute (NHLBI), National Institutes of Health, Bethesda, Md

**Keywords:** Adults, asthma, asthma triggers, cluster analysis, emergency department, disparities, patient-reported, social determinants of health, survey

## Abstract

**Background:**

Social determinants of health (SDOH) and environmental triggers contribute to risk of asthma exacerbations. Electronic health record data rarely capture complete SDOH and asthma trigger information, thereby limiting comprehensive risk factor assessments absent additional data sources.

**Objective:**

We collected detailed SDOH and trigger data from patients with asthma who were identified from electronic health records to better understand modifiable risk factors contributing to risk of asthma emergency department (ED) visits.

**Methods:**

We invited patients with asthma identified through the Penn Medicine electronic health record to complete an online questionnaire covering SDOH, asthma triggers, and asthma history. Zero-inflated Poisson regression models were fit to identify factors associated with ED visits for asthma, and hierarchical agglomerative clustering was used to explore underlying patterns in the data.

**Results:**

Among 974 survey respondents, 206 reported one or more asthma-related ED visit in the last year. Nearly all SDOH and asthma trigger variables were significantly associated with asthma ED visits in univariable models, but most of these effects were strongly attenuated in multivariable regression models. The effect of race, however, remained strong in both models. *Post hoc* analyses showed that additionally adjusting for SDOH variables attenuated the relationships between asthma triggers and ED visits. Cluster analysis revealed that the subgroup of participants reporting highest social vulnerability and most asthma triggers also had the most asthma-related ED visits, hospitalizations, and days missed of work or school (all *P* < 10^−4^).

**Conclusion:**

Self-reported SDOH and asthma trigger data are useful to identify subpopulations at highest risk for adverse asthma outcomes.

## Visual Abstract

**Figure:**
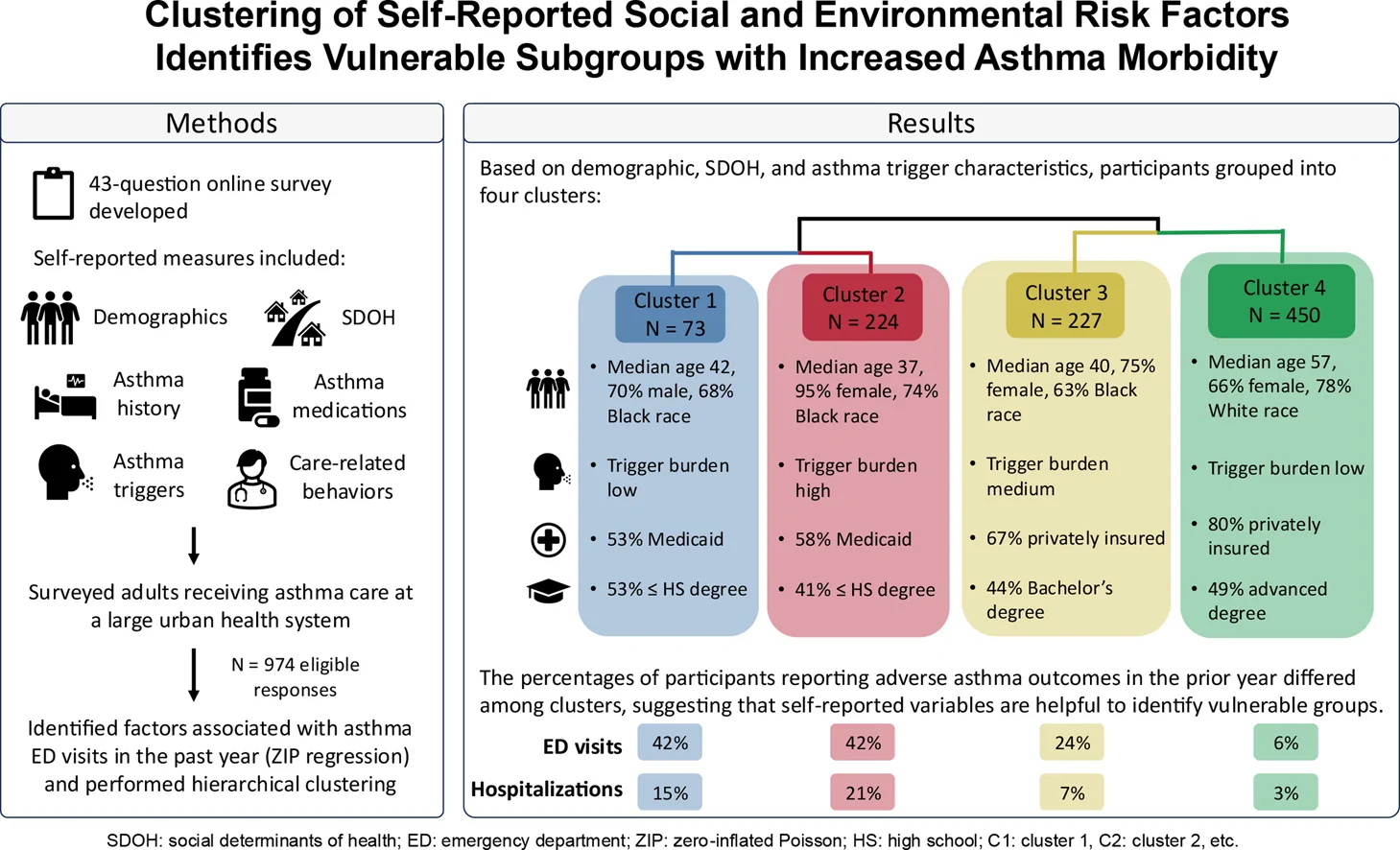

Asthma exacerbations, defined as episodes of worsening symptoms, remain common in some patients despite the availability of effective guideline-directed clinical asthma management strategies.^1^^,^^2^ Exacerbations affect patients of all asthma severity levels^3^ and contribute to increased asthma-related morbidity and mortality as well as increased health care costs.^4^^,^^5^ Personalized action plans to address modifiable risk factors, including identifying and avoiding asthma triggers, are therefore a key component of asthma management.^1^^,^^6^ Common factors known to trigger asthma exacerbations (hereafter referred to as *asthma triggers*) include environmental exposures such as allergens,^7^^,^^8^ viruses,^9^^,^^10^ bacteria,^11^^,^^12^ and air pollutants;^13^ physical activity;^14^^,^^15^ and psychological factors.^16^^,^^17^

Asthma exacerbations have well-documented associations with many social determinants of health (SDOH) that are not easily modifiable, including living in poverty,^18^^,^^19^ substandard housing conditions,^20^^,^^21^ exposure to violence,^22^^,^^23^ and limited access to health care.^24^^,^^25^ The SDOH that indicate social vulnerability also increase likelihood of exposure to common asthma triggers. Furthermore, racial and ethnic disparities in asthma burden remain a persistent problem in the United States.^26^, ^27^, ^28^, ^29^, ^30^ Because minoritized race and ethnicity are highly correlated with various SDOH and harmful environmental exposures that differ across the country and within regions, asthma disparities remain poorly understood and difficult to address without regional and personalized strategies.^31^

At the level of health care systems, electronic health records (EHRs) offer an opportunity to better understand patterns of asthma risk factors within a health system. However, EHR data rarely capture complete SDOH and asthma trigger information for patients, limiting their ability to provide comprehensive risk factor assessments. Improving the characterization of social determinants of asthma in EHRs and incorporating this knowledge into asthma management practices offers a cost-effective strategy to decrease the burden of asthma.^31^^,^^32^ Here, we surveyed adults with asthma from Penn Medicine, a large multihospital health system, on their asthma history, asthma triggers, and SDOH, aiming to improve our understanding of modifiable asthma risk factors that contribute to asthma disparities.

## Methods

We invited adults with asthma seen at Penn Medicine to complete a 20- to 30-minute online questionnaire that assessed participants’ asthma history, SDOH measures, and asthma triggers. The survey contained 43 questions, which were adapted from questionnaires reported by the PhenX Toolkit,^33^ the Asthma Trigger Inventory,^34^ and a clinical trial protocol.^35^ Our study was approved by the University of Pennsylvania institutional review board under protocol 824789. Additional methodological details can be found in the Methods section in the Online Repository available at www.jaci-global.org.

### Study population and survey administration

Participants were assessed for eligibility using Penn Medicine EHR data. Adults (age ≥ 18 years) who had at least one encounter with an International Classification of Diseases 10 code for asthma (J45∗) and who were prescribed a short-acting β_2_-agonist were invited to complete the survey. Patients were approached in batches between June 2023 and May 2024 until we reached our target enrollment of 1,000 respondents with a demographic distribution in terms of sex and race/ethnicity similar to the overall Penn Medicine asthma patient population. Participants who completed the survey received a $30 gift card as payment.

### Survey measures

Demographic variables included participants’ age, sex, race, and ethnicity. Health factors that have been associated with asthma were measured, including smoking history, body mass index (BMI), and comorbid chronic obstructive pulmonary disease (COPD). Participants were asked about their number of asthma-related emergency department (ED) visits, overnight hospital stays, intensive care unit visits, and intubations in the last 12 months, as well as whether they experienced neighborhood violence, housing insecurity, food insecurity, transportation insecurity, energy insecurity (ie, lack of access to electricity/gas), or communication insecurity (ie, lack of access to telephone/cell phone service). Participants were also asked about their health insurance type and educational attainment, and to rate whether 28 common triggers worsened their asthma symptoms on a 5-point Likert scale. Other questions measured participants’ asthma history, current medication receipt, and care-related behaviors. Survey items consisted of a mixture of Likert-scale, multiple-choice, and open-ended questions. Additional information is provided in the Methods section in the Online Repository.

### Statistical analysis

All analyses were conducted in R v4.2 software.^36^ The primary outcome variable was the number of self-reported ED visits in the last 12 months. As a result of varying amounts of nonresponse in ED visits (31 responses were missing) and independent variables (captured as unknown level for categorical variables; see the Methods section in the Online Repository), sample sizes varied for individual analyses (n = 839-974). Chi-square and Kruskal-Wallis rank sum tests were used in bivariate analyses to compare the characteristics of nonrespondents with study participants, and to compare the characteristics of complete cases versus participants with missing data.

### Asthma trigger subscale scores

Following the methods described by Ritz et al,^34^ principal component analysis (PCA) with orthogonal varimax rotation was used to reduce the dimensionality of the trigger data. Nonmissing trigger items within each PCA factor were averaged to create 5 continuous variables, hereafter referred to as *trigger subscale scores.* These scores ranged 0 to 4, with 0 indicating that items within the subscale never trigger asthma symptoms and 4 indicating that those items always trigger asthma symptoms.

### Zero-inflated poisson regression models

Univariable and multivariable zero-inflated Poisson regression (ZIP) models were fit with ED visits as a count (0, 1, 2, 3, 4+) outcome and with demographics, comorbidities, trigger subscales, and SDOH variables as predictors. Additional multivariable ZIP regression models were fit with age modeled as a natural cubic spline, and the results were used to derive a categorical age variable. Relationships with *P* < .05 were considered statistically significant.

### Variable multicollinearity

We checked for multicollinearity by computing the Pearson correlation coefficients between all variables included in the multivariable model, using one-hot encoding for nominal categorical variables.

### Hierarchical multiple regression models

*Post hoc* analyses were run to better understand the relationships among trigger subscale scores, SDOH, and ED visits. Two-step hierarchical ZIP regression models were fit with ED visits as the outcome while adjusting for each trigger subscale one at a time. For each of these models, age, sex, and BMI were included as covariates in step 1, and the 8 SDOH variables were added to each model in step 2. As a sensitivity analysis, we repeated the regression analyses with race included as an additional covariate in step 1. Additionally, linear regression models were fit to further explore associations between each trigger subscale score as a continuous outcome while including all 8 SDOH variables, age, sex, and BMI as independent variables.

### Hierarchical agglomerative clustering

As an alternative analysis to identify distinct subgroups of patients with asthma according to their demographic characteristics, asthma triggers, and SDOH responses, the Ward minimum-variance method with an agglomerative approach was used to perform hierarchical clustering of participants. Variables included in the clustering were the independent variables used in the ZIP regression. Silhouette scores were computed to determine the optimal number of clusters.^37^ Chi-square and Kruskal-Wallis rank sum tests were used to assess the between-cluster distribution of included variables and the between-cluster distribution of held-out variables.

## Results

### Survey administration

A diagram showing the number of people at each stage of the survey procedure is provided in Fig E1 in this article’s Online Repository available at www.jaci-global.org. Of the 6,510 contacted participants, 974 of the responses we received were eligible for analysis. Recruitment rate and survey completion were higher in respondents who were female and White (see Table E1 in the Online Repository). The distribution of study participants’ sex and race/ethnicity reflected that of the eligible Penn Medicine adults with asthma.

### Asthma trigger subscale analysis

PCA of the 28 self-reported asthma triggers resulted in a 5-factor solution explaining 71% of the variance in individual triggers (see Table E2 in the Online Repository available at www.jaci-global.org). On the basis of the distribution of these loadings between factors, each factor was found to correspond to a trigger theme identified by Ritz et al.^34^ The themes and corresponding percentage variance in individual triggers explained by each respective theme were air pollution/irritant triggers (12%), psychological triggers (23%), allergen triggers (10%), physical activity triggers (11%), and infection triggers (15%).

### Participant characteristics

Of the 974 study participants, 737 reported no asthma-related ED visits in the past year and 206 reported one or more ED visits (Table I). Participants who reported more ED visits in the past year were more likely to be of Black race, be a current smoker or have passive smoke exposure, have a higher BMI, and have COPD. Overall, mean (standard deviation [SD]) trigger subscale scores were lowest for psychological triggers, at 0.68 (0.89), and highest for infection triggers, at 2.23 (1.25), indicating that participants reported psychological triggers less frequently and infection triggers more frequently overall. Trigger subscale scores increased proportionally with ED visits, such that participants with no ED visits in the last year reported psychological triggers corresponding to a mean (SD) subscale score of 0.56 (0.79), compared to 1.78 (1.26) for participants with 4+ visits last year. The number of participants reporting higher social risk according to measured SDOH variables related to socioeconomic status, neighborhood deprivation, and exposure to violence, hereafter referred to as having *higher social vulnerability,* also increased proportionately with ED visits. Most notably, 23% of participants with no ED visits in the last year reported food insecurity, compared to 83% of participants with 4+ visits. Univariable analyses comparing the characteristics of complete cases versus participants excluded because of incomplete survey data found the greatest differences in age, smoking history, air pollution/irritant triggers, allergen triggers, education, and transportation insecurity (*P* < 10^−4^) (see Table E3 in the Online Repository available at www.jaci-global.org). Nonresponse rates for all independent variables are shown in Table I.

**Table I:** Participant characteristics by ED visits

Characteristic	Overall (N = 974)	No. of ED visits in past year					
		0 (n = 737)	1 (n = 84)	2 (n = 64)	3 (n = 29)	4+ (n = 29)	Unknown (n = 31)
**Demographics and comorbidities**							
Age (years)	47 (34, 61)	49 (36, 63)	38 (31, 52)	34 (31, 49)	39 (30, 50)	43 (33, 53)	55 (42, 66)
Sex							
Male	273 (28%)	212 (29%)	22 (26%)	14 (22%)	7 (24%)	9 (31%)	9 (29%)
Female	701 (72%)	525 (71%)	62 (74%)	50 (78%)	22 (76%)	20 (69%)	22 (71%)
Race and ethnicity							
American Indian or Alaskan Native	6 (0.6%)	2 (0.3%)	1 (1.2%)	2 (3.1%)	1 (3.4%)	0	0
Asian	51 (5.2%)	43 (5.8%)	5 (6.0%)	3 (4.7%)	0	0	0
Black	393 (40%)	235 (32%)	53 (63%)	43 (67%)	21 (72%)	19 (66%)	22 (71%)
Hispanic or Latino	33 (3.4%)	22 (3.0%)	6 (7.1%)	2 (3.1%)	0	2 (6.9%)	1 (3.2%)
White	399 (41%)	362 (49%)	14 (17%)	8 (13%)	4 (14%)	4 (14%)	7 (23%)
Middle Eastern or North African	6 (0.6%)	6 (0.8%)	0	0	0	0	0
Native Hawaiian or Pacific Islander	0	0	0	0	0	0	0
Multiracial	71 (7.3%)	54 (7.3%)	5 (6.0%)	6 (9.4%)	2 (6.9%)	3 (10%)	1 (3.2%)
Unknown	15 (1.5%)	13 (1.8%)	0	0	1 (3.4%)	1 (3.4%)	0
Smoking history							
None	612 (63%)	478 (65%)	55 (65%)	34 (53%)	19 (66%)	15 (52%)	11 (35%)
Current	60 (6.2%)	30 (4.1%)	4 (4.8%)	12 (19%)	4 (14%)	4 (14%)	6 (19%)
Past	238 (24%)	185 (25%)	19 (23%)	12 (19%)	4 (14%)	6 (21%)	12 (39%)
Passive	52 (5.3%)	34 (4.6%)	6 (7.1%)	6 (9.4%)	2 (6.9%)	4 (14%)	0
Unknown	12 (1.2%)	10 (1.4%)	0	0	0	0	2 (6.5%)
BMI							
Not overweight or obese	239 (25%)	203 (28%)	15 (18%)	12 (19%)	3 (10%)	4 (14%)	2 (6.5%)
Overweight	277 (28%)	222 (30%)	16 (19%)	14 (22%)	11 (38%)	4 (14%)	10 (32%)
Class 1 obesity	188 (19%)	137 (19%)	19 (23%)	14 (22%)	3 (10%)	7 (24%)	8 (26%)
Class 2 obesity	121 (12%)	76 (10%)	11 (13%)	13 (20%)	5 (17%)	13 (45%)	3 (9.7%)
Class 3 obesity	131 (13%)	84 (11%)	22 (26%)	10 (16%)	7 (24%)	1 (3.4%)	7 (23%)
Unknown	18 (1.8%)	15 (2.0%)	1 (1.2%)	1 (1.6%)	0	0	1 (3.2%)
COPD							
No	828 (85%)	636 (86%)	74 (88%)	50 (78%)	24 (83%)	21 (72%)	23 (74%)
Yes	97 (10.0%)	66 (9.0%)	8 (9.5%)	9 (14%)	2 (6.9%)	8 (28%)	4 (13%)
Unknown	49 (5.0%)	35 (4.7%)	2 (2.4%)	5 (7.8%)	3 (10%)	0	4 (13%)
**Trigger subscale scores**							
Psychological	0.68 (0.89)	0.56 (0.79)	0.78 (0.87)	1.29 (1.05)	0.92 (1.06)	1.78 (1.26)	0.93 (1.02)
Unknown	73	55	6	6	2	0	4
Air pollution/irritants	1.59 (1.13)	1.49 (1.10)	1.69 (1.06)	2.03 (1.19)	1.77 (1.15)	2.54 (1.22)	1.69 (1.23)
Unknown	52	41	5	2	2	0	2
Physical activity	1.73 (1.17)	1.64 (1.16)	1.79 (1.14)	2.24 (1.18)	1.94 (1.13)	2.34 (1.16)	1.70 (1.22)
Unknown	45	29	7	2	2	0	5
Allergens	1.81 (1.14)	1.71 (1.11)	1.93 (1.18)	2.21 (1.09)	2.14 (1.17)	2.57 (1.04)	1.82 (1.33)
Unknown	31	24	1	3	1	0	2
Infection	2.23 (1.25)	2.11 (1.24)	2.44 (1.14)	2.73 (1.14)	2.70 (1.19)	3.15 (1.00)	2.05 (1.36)
Unknown	29	23	2	2	0	0	2
**SDOH**							
Health insurance type							
Private	585 (60%)	496 (67%)	43 (51%)	21 (33%)	10 (34%)	4 (14%)	11 (35%)
Medicaid	227 (23%)	119 (16%)	29 (35%)	28 (44%)	15 (52%)	22 (76%)	14 (45%)
Medicare	115 (12%)	87 (12%)	9 (11%)	11 (17%)	1 (3.4%)	2 (6.9%)	5 (16%)
Military	5 (0.5%)	4 (0.5%)	0	1 (1.6%)	0	0	0
None	14 (1.4%)	8 (1.1%)	1 (1.2%)	1 (1.6%)	2 (6.9%)	1 (3.4%)	1 (3.2%)
Other	6 (0.6%)	4 (0.5%)	0	2 (3.1%)	0	0	0
Unknown	22 (2.3%)	19 (2.6%)	2 (2.4%)	0	1 (3.4%)	0	0
Education							
High school or below	198 (20%)	114 (15%)	20 (24%)	31 (48%)	8 (28%)	13 (45%)	12 (39%)
Some college	242 (25%)	158 (21%)	30 (36%)	20 (31%)	14 (48%)	10 (34%)	10 (32%)
Bachelor	258 (26%)	222 (30%)	17 (20%)	7 (11%)	5 (17%)	4 (14%)	3 (9.7%)
Advanced degree	268 (28%)	237 (32%)	17 (20%)	6 (9.4%)	2 (6.9%)	2 (6.9%)	4 (13%)
Unknown	8 (0.8%)	6 (0.8%)	0	0	0	0	2 (6.5%)
Housing insecurity							
No	914 (94%)	710 (96%)	78 (93%)	53 (83%)	25 (86%)	22 (76%)	26 (84%)
Yes	36 (3.7%)	13 (1.8%)	5 (6.0%)	9 (14%)	2 (6.9%)	5 (17%)	2 (6.5%)
Unknown	24 (2.5%)	14 (1.9%)	1 (1.2%)	2 (3.1%)	2 (6.9%)	2 (6.9%)	3 (9.7%)
Food insecurity							
No	672 (69%)	567 (77%)	47 (56%)	23 (36%)	14 (48%)	5 (17%)	16 (52%)
Yes	300 (31%)	169 (23%)	37 (44%)	41 (64%)	15 (52%)	24 (83%)	14 (45%)
Unknown	2 (0.2%)	1 (0.1%)	0	0	0	0	1 (3.2%)
Transportation insecurity							
No	834 (86%)	667 (91%)	70 (83%)	46 (72%)	18 (62%)	10 (34%)	23 (74%)
Yes	134 (14%)	66 (9.0%)	14 (17%)	18 (28%)	11 (38%)	19 (66%)	6 (19%)
Unknown	6 (0.6%)	4 (0.5%)	0	0	0	0	2 (6.5%)
Energy insecurity							
No	913 (94%)	709 (96%)	77 (92%)	53 (83%)	24 (83%)	21 (72%)	29 (94%)
Yes	54 (5.5%)	26 (3.5%)	6 (7.1%)	10 (16%)	4 (14%)	7 (24%)	1 (3.2%)
Unknown	7 (0.7%)	2 (0.3%)	1 (1.2%)	1 (1.6%)	1 (3.4%)	1 (3.4%)	1 (3.2%)
Communication insecurity							
No	892 (92%)	697 (95%)	74 (88%)	50 (78%)	24 (83%)	21 (72%)	26 (84%)
Yes	74 (7.6%)	35 (4.7%)	9 (11%)	13 (20%)	5 (17%)	8 (28%)	4 (13%)
Unknown	8 (0.8%)	5 (0.7%)	1 (1.2%)	1 (1.6%)	0	0	1 (3.2%)
Exposure to violence							
No	793 (81%)	627 (85%)	64 (76%)	42 (66%)	21 (72%)	16 (55%)	23 (74%)
Yes	171 (18%)	105 (14%)	17 (20%)	22 (34%)	8 (28%)	13 (45%)	6 (19%)
Unknown	10 (1.0%)	5 (0.7%)	3 (3.6%)	0	0	0	2 (6.5%)

For entire study population and for each ED visit count, no. (%) of participants is shown for categorical variables; mean (SD) for continuous variables; and median (IQR) for discrete variables.

### Factors associated with ED visits

The incidence rate ratios (IRRs) for all variables included in univariable and multivariable ZIP regression are summarized in Table II. In univariable ZIP regression analyses, Black race (IRR = 3.87; 95% confidence interval [CI], 2.71, 5.50) and class 2 obesity (IRR = 2.13; 95% CI, 1.43, 3.15) were most positively associated with having a higher count of ED visits in the last year, as were all 5 trigger subscale scores and many SDOH variables (*P* < .001). Having a bachelor’s or advanced degree was negatively associated with ED visits (*P* < .001). Although Pearson correlation analysis revealed moderate positive correlations between pairs of trigger subscales and weak positive correlations between trigger subscales and SDOH variables (see Fig E2 in the Online Repository available at www.jaci-global.org), including all variables in a multivariable ZIP regression model did not result in inflated standard errors, suggesting that it was appropriate to include all variables simultaneously. In the multivariable model, the effects observed in univariable regression models were strongly attenuated, with only Black race remaining strongly (*P* < .001) associated with ED visits, although with a decreased IRR of 2.26 (95% CI, 1.50, 3.41).

**Table II:** Asthma ED visit risk factors in univariable and multivariable ZIP regression models

Characteristic	Univariable				Multivariable			
	No.	Unadjusted IRR	95% CI	*P*	No.	Adjusted IRR	95% CI	*P*
**Demographics and comorbidities**								
Age	924				839			
18-44 years		—	—			—	—	
45-64 years		0.94	0.72, 1.23	.67		1.02	0.74, 1.40	.92
65+ years		0.59	0.37, 0.93	.023		1.28	0.77, 2.12	.34
Sex	924				839			
Male		—	—			—	—	
Female		1.01	0.78, 1.32	.92		0.77	0.57, 1.04	.084
Race and ethnicity	924				839			
White		—	—			—	—	
Asian		1.31	0.62, 2.77	.48		0.98	0.45, 2.15	.97
Black		3.87	2.71, 5.50	<10^−4^		2.26	1.50, 3.41	<10^−4^
Multiracial		3.51	2.08, 5.92	<10^−4^		1.80	1.04, 3.10	.035
Unknown/other		3.49	2.08, 5.87	<10^−4^		1.94	1.08, 3.48	.026
Smoking history	924				839			
None		—	—			—	—	
Current		1.51	1.09, 2.08	.013		1.01	0.67, 1.52	.97
Past		0.92	0.67, 1.26	.61		0.90	0.65, 1.26	.54
Passive		1.38	0.95, 2.01	.095		0.83	0.54, 1.28	.40
BMI	924				839			
Not overweight or obese		—	—			—	—	
Overweight		1.32	0.87, 1.99	.19		0.91	0.61, 1.35	.62
Class 1 obesity		1.44	0.95, 2.17	.084		1.18	0.78, 1.78	.43
Class 2 obesity		2.13	1.43, 3.15	1.7 × 10^−4^		1.44	0.95, 2.17	.085
Class 3 obesity		1.29	0.85, 1.98	.23		1.19	0.76, 1.87	.45
Unknown		0.58	0.14, 2.32	.44		0.16	0.02, 1.32	.089
COPD	924				839			
No		—	—			—	—	
Yes		1.42	1.05, 1.93	0.023		1.42	0.97, 2.08	.075
Unknown		1.13	0.66, 1.91	0.66		0.91	0.49, 1.68	.76
Trigger subscale scores								
Psychological	858	1.35	1.22, 1.50	<10^−4^	839	1.17	0.99, 1.37	.061
Air pollution/irritants	875	1.26	1.14, 1.39	<10^−4^	839	0.99	0.83, 1.19	.93
Physical activity	886	1.19	1.07, 1.32	8.6 × 10^−4^	839	0.89	0.77, 1.04	.15
Allergens	896	1.25	1.12, 1.39	<10^−4^	839	1.15	0.97, 1.36	.11
Infection	898	1.33	1.19, 1.48	<10^−4^	839	1.15	0.99, 1.33	.066
**SDOH**								
Health insurance type	924				839			
Private		—	—			—	—	
Medicaid		2.99	2.24, 4.00	<10^−4^		1.35	0.98, 1.87	.067
Medicare		1.66	1.07, 2.55	.023		1.09	0.67, 1.78	.72
Unknown/other		2.37	1.38, 4.07	.0017		1.98	1.11, 3.54	.021
Education	924				839			
High school or below		—	—			—	—	
Some college		0.88	0.68, 1.13	.32		1.27	0.96, 1.69	.096
Bachelor		0.41	0.28, 0.60	<10^−4^		0.88	0.58, 1.33	.54
Advanced degree		0.25	0.17, 0.38	<10^−4^		0.69	0.43, 1.12	.13
Housing insecurity	924				839			
No		—	—			—	—	
Yes		1.56	1.13, 2.15	.0064		0.92	0.59, 1.42	.70
Unknown		1.76	1.06, 2.91	.027		2.43	1.05, 5.63	.039
Food insecurity	924				839			
No		—	—			—	—	
Yes		2.67	2.03, 3.52	<10^−4^		1.47	1.08, 2.02	.015
Transportation insecurity	924				839			
No		—	—			—	—	
Yes		2.25	1.75, 2.88	<10^−4^		1.32	0.96, 1.82	.082
Energy insecurity	924				839			
No		—	—			—	—	
Yes		1.64	1.23, 2.20	7.7 × 10^−4^		1.07	0.71, 1.61	.75
Unknown		1.71	0.90, 3.28	.10		2.14	1.02, 4.47	.043
Communication insecurity	924				839			
No		—	—			—	—	
Yes		1.56	1.19, 2.04	.0013		1.01	0.70, 1.44	.98
Unknown		0.81	0.22, 2.96	.75		0.63	0.17, 2.31	.49
Exposure to violence	924				839			
No		—	—			—	—	
Yes		1.54	1.21, 1.97	4.4 × 10^−4^		1.12	0.85, 1.48	.42
Unknown		0.64	0.19, 2.12	.46		0.41	0.09, 1.87	.25

ZIP regression models of asthma-related ED visits as count outcome.

### Relationships between individual trigger subscale scores and ED visits

Given the moderate positive correlations between trigger subscale scores, 2-step hierarchical ZIP regression models were created as an alternative analysis to assess the relationship of each trigger subscale score to ED visits while introducing other independent variables in stages. While adjusting for age, sex, and BMI but not for SDOH variables, each trigger subscale score was significantly positively associated with having a higher count of ED visits in the last year, although psychological triggers (IRR = 1.42; 95% CI, 1.29, 1.43) and infection triggers (IRR = 1.40; 95% CI, 1.26 1.56) had the strongest positive associations (*P* < 10^−4^) (Table III). Additionally adjusting for SDOH variables attenuated the IRRs for all trigger subscale scores, and only psychological (IRR = 1.29; 95% CI, 1.16, 1.43), allergen (IRR = 1.22; 95% CI, 1.11, 1.35), and infection triggers (IRR = 1.28; 95% CI, 1.16, 1.42) remained significantly positively associated with ED visits (*P* < 10^−4^). This attenuation was still observed in sensitivity analyses that adjusted for race and ethnicity (see Table E4 in the Online Repository available at www.jaci-global.org).

**Table III:** Associations between individual trigger subscales and ED visits with and without adjusting for SDOH variables

Trigger subscale	Unadjusted for SDOH				Adjusted for SDOH			
	No.	Adjusted IRR	95% CI	*P*	No.	Adjusted IRR	95% CI	*P*
Psychological	863	1.42	1.30, 1.56	<10^−4^	863	1.29	1.16, 1.43	<10^−4^
Air pollution/irritants	882	1.27	1.16, 1.40	<10^−4^	882	1.17	1.06, 1.28	.0014
Physical activity	892	1.25	1.14, 1.37	<10^−4^	892	1.10	1.0, 1.21	.064
Allergens	903	1.27	1.16, 1.40	<10^−4^	903	1.22	1.11, 1.35	<10^−4^
Infection	905	1.40	1.26, 1.56	<10^−4^	905	1.28	1.16, 1.42	<10^−4^

Hierarchical ZIP regression models of ED visits as count outcome. Each row corresponds to ZIP model adjusted for 1 of 5 trigger subscale scores with age, sex, and BMI included as covariates in step 1 (unadjusted for SDOH). All 8 SDOH variables were then added to each model as additional covariates in step 2 (adjusted for SDOH).

### Relationships between individual trigger subscale scores and SDOH variables

Five linear regression models, each fit with one trigger subscale score as the outcome, found significant associations between several SDOH variables and trigger subscale scores (see Table E5 in the Online Repository available at www.jaci-global.org), the strongest of which were for Medicaid versus private insurance increasing psychological trigger subscale score (β = 0.33; 95% CI, 0.17, 0.49) and for having food insecurity increasing air pollution/irritants trigger subscale score (β = 0.39; 95% CI, 0.20, 0.58) (*P* < 10^−4^).

### Clusters of participants according to survey responses

**Fig 1:**
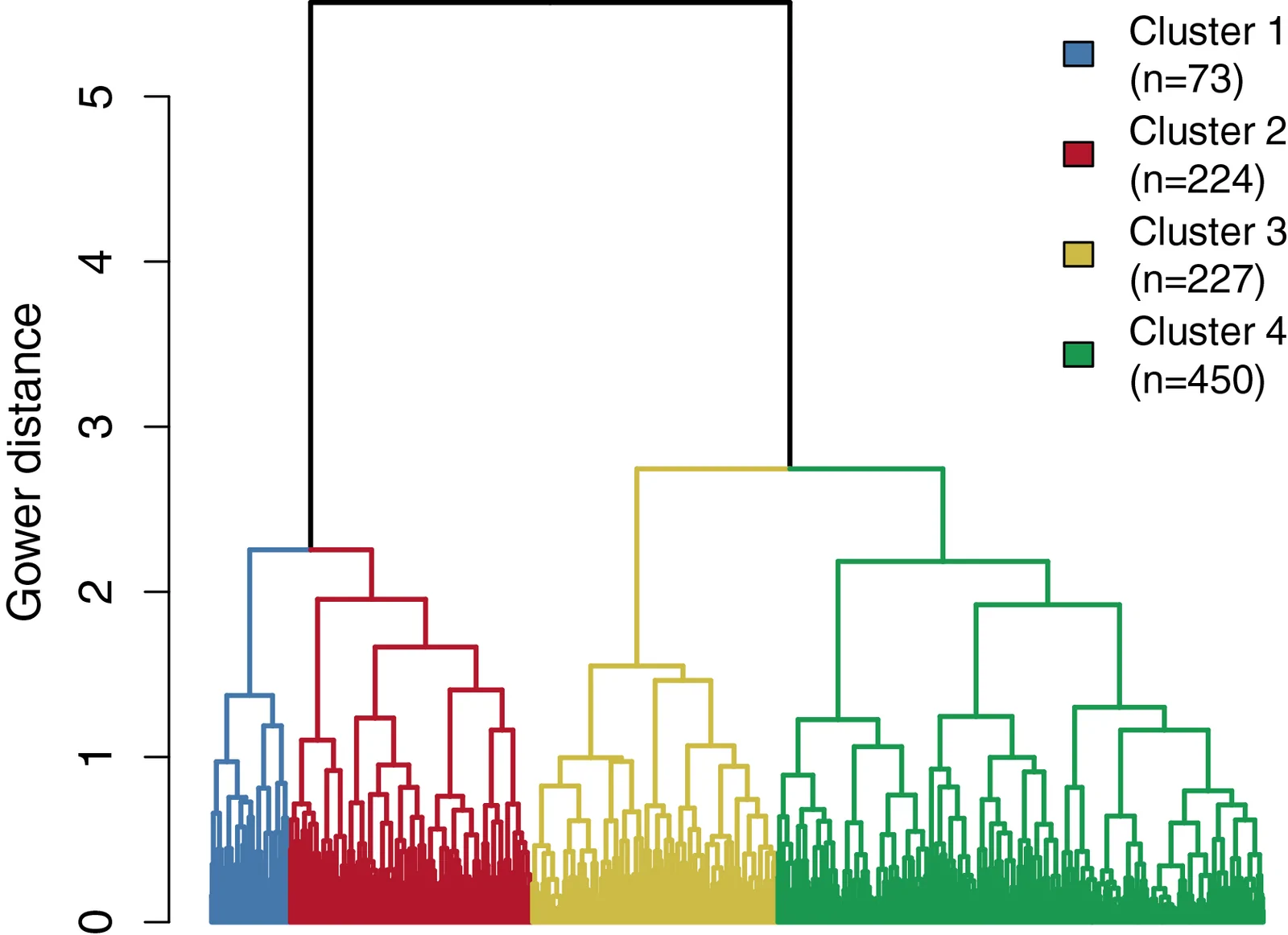
Dendrogram resulting from hierarchical agglomerative clustering of observations. Clustering was performed by Ward minimum-variance method. Pairwise dissimilarity was computed by Gower distance based on self-reported participant demographics, comorbidities, asthma triggers, and SDOH. Dendrogram was cut based on silhouette width so that 4 clusters emerged.

A dendrogram of hierarchical agglomerative clustering results that were based on demographic, trigger subscale score, and SDOH variables is shown in Fig 1. On the basis of silhouette width, the dendrogram was cut into 4 clusters: cluster 1 (n = 73), cluster 2 (n = 224), cluster 3 (n = 227), and cluster 4 (n = 450). To better understand which variables contributed to the clustering, we compared the distributions of clustering variables across the 4 clusters as shown in Table IV. Clusters differed significantly (*P* < 10^−4^) by all demographic and comorbidity variables except COPD. Cluster 1 had the highest percentage of male participants (70%) and of participants reporting the most past or current smoking (38% and 21%, respectively). Cluster 2 had the highest proportion of participants reporting class 2 or 3 obesity (17% and 26%, respectively). Clusters 1, 2, and 3 had higher proportions of participants of Black race (68%, 74%, 63%, respectively) than cluster 4 (7.8%). Cluster 4 participants were older, with a median (interquartile range [IQR]) age of 57 (43-68) years.

**Table IV:** Participant characteristics by clustering variables

Characteristic	Overall (N = 974)	Cluster				*P*
		1 (n = 73)	2 (n = 224)	3 (n = 227)	4 (n = 450)	
**Demographics and comorbidities**						
Age (years)	47 (34, 61)	42 (32, 54)	37 (30, 50)	40 (32, 54)	57 (43, 68)	<10^−4^
Sex						<10^−4^
Male	273 (28%)	51 (70%)	12 (5.4%)	57 (25%)	153 (34%)	
Female	701 (72%)	22 (30%)	212 (95%)	170 (75%)	297 (66%)	
Race and ethnicity						<10^−4^
Asian	51 (5.2%)	4 (5.5%)	4 (1.8%)	19 (8.4%)	24 (5.3%)	
Black	393 (40%)	50 (68%)	166 (74%)	142 (63%)	35 (7.8%)	
White	399 (41%)	9 (12%)	13 (5.8%)	26 (11%)	351 (78%)	
Multiracial	71 (7.3%)	8 (11%)	21 (9.4%)	28 (12%)	14 (3.1%)	
Unknown/other	60 (6.2%)	2 (2.7%)	20 (8.9%)	12 (5.3%)	26 (5.8%)	
Smoking history						<10^−4^
None	612 (63%)	28 (38%)	131 (58%)	177 (78%)	276 (61%)	
Current	60 (6.2%)	15 (21%)	28 (13%)	9 (4.0%)	8 (1.8%)	
Past	238 (24%)	28 (38%)	38 (17%)	21 (9.3%)	151 (34%)	
Passive	52 (5.3%)	2 (2.7%)	25 (11%)	18 (7.9%)	7 (1.6%)	
Unknown	12 (1.2%)	0	2 (0.9%)	2 (0.9%)	8 (1.8%)	
BMI						<10^−4^
Not overweight or obese	239 (25%)	12 (16%)	24 (11%)	60 (26%)	143 (32%)	
Overweight	277 (28%)	26 (36%)	53 (24%)	37 (16%)	161 (36%)	
Class 1 obesity	188 (19%)	20 (27%)	48 (21%)	58 (26%)	62 (14%)	
Class 2 obesity	121 (12%)	4 (5.5%)	38 (17%)	32 (14%)	47 (10%)	
Class 3 obesity	131 (13%)	9 (12%)	59 (26%)	33 (15%)	30 (6.7%)	
Unknown	18 (1.8%)	2 (2.7%)	2 (0.9%)	7 (3.1%)	7 (1.6%)	
COPD						.022
No	828 (85%)	57 (78%)	188 (84%)	209 (92%)	374 (83%)	
Yes	97 (10.0%)	11 (15%)	24 (11%)	9 (4.0%)	53 (12%)	
Unknown	49 (5.0%)	5 (6.8%)	12 (5.4%)	9 (4.0%)	23 (5.1%)	
Trigger subscale scores						
Psychological	0.68 (0.89)	0.52 (0.67)	1.32 (1.08)	0.67 (0.88)	0.39 (0.62)	<10^−4^
Air pollution/irritants	1.59 (1.13)	1.05 (0.94)	2.25 (0.98)	1.67 (1.09)	1.30 (1.11)	<10^−4^
Physical activity	1.73 (1.17)	1.10 (0.96)	2.48 (0.94)	1.96 (1.12)	1.34 (1.11)	<10^−4^
Allergens	1.81 (1.14)	1.20 (1.03)	2.49 (0.99)	1.97 (1.09)	1.48 (1.07)	<10^−4^
Infection	2.23 (1.25)	1.67 (1.27)	3.02 (0.92)	2.39 (1.06)	1.85 (1.27)	<10^−4^
**SDOH**						
Health insurance type						<10^−4^
Private	585 (60%)	14 (19%)	62 (28%)	151 (67%)	358 (80%)	
Medicaid	227 (23%)	39 (53%)	131 (58%)	45 (20%)	12 (2.7%)	
Medicare	115 (12%)	15 (21%)	21 (9.4%)	21 (9.3%)	58 (13%)	
Unknown/other	47 (4.8%)	5 (6.8%)	10 (4.5%)	10 (4.4%)	22 (4.9%)	
Education						<10^−4^
High school or below	198 (20%)	39 (53%)	91 (41%)	38 (17%)	30 (6.7%)	
Some college	242 (25%)	24 (33%)	74 (33%)	69 (30%)	75 (17%)	
Bachelor	258 (26%)	3 (4.1%)	38 (17%)	101 (44%)	116 (26%)	
Advanced degree	268 (28%)	7 (9.6%)	21 (9.4%)	18 (7.9%)	222 (49%)	
Unknown	8 (0.8%)	0	0	1 (0.4%)	7 (1.6%)	
Housing insecurity						<10^−4^
No	914 (94%)	61 (84%)	189 (84%)	222 (98%)	442 (98%)	
Yes	36 (3.7%)	7 (9.6%)	26 (12%)	3 (1.3%)	0	
Unknown	24 (2.5%)	5 (6.8%)	9 (4.0%)	2 (0.9%)	8 (1.8%)	
Food insecurity						<10^−4^
No	672 (69%)	12 (16%)	30 (13%)	216 (95%)	414 (92%)	
Yes	300 (31%)	61 (84%)	194 (87%)	10 (4.4%)	35 (7.8%)	
Unknown	2 (0.2%)	0	0	1 (0.4%)	1 (0.2%)	
Transportation insecurity						<10^−4^
No	834 (86%)	50 (68%)	122 (54%)	221 (97%)	441 (98%)	
Yes	134 (14%)	23 (32%)	100 (45%)	5 (2.2%)	6 (1.3%)	
Unknown	6 (0.6%)	0	2 (0.9%)	1 (0.4%)	3 (0.7%)	
Energy insecurity						<10^−4^
No	913 (94%)	61 (84%)	193 (86%)	219 (96%)	440 (98%)	
Yes	54 (5.5%)	12 (16%)	29 (13%)	7 (3.1%)	6 (1.3%)	
Unknown	7 (0.7%)	0	2 (0.9%)	1 (0.4%)	4 (0.9%)	
Communication insecurity						<10^−4^
No	892 (92%)	56 (77%)	173 (77%)	220 (97%)	443 (98%)	
Yes	74 (7.6%)	14 (19%)	50 (22%)	7 (3.1%)	3 (0.7%)	
Unknown	8 (0.8%)	3 (4.1%)	1 (0.4%)	0	4 (0.9%)	
Exposure to violence						<10^−4^
No	793 (81%)	53 (73%)	136 (61%)	186 (82%)	418 (93%)	
Yes	171 (18%)	19 (26%)	85 (38%)	39 (17%)	28 (6.2%)	
Unknown	10 (1.0%)	1 (1.4%)	3 (1.3%)	2 (0.9%)	4 (0.9%)	

Shown are no. (%) of participants for categorical variables; mean (SD) for continuous variables; and median (IQR) for discrete variables across clusters. Characteristics correspond to variables used as inputs in hierarchical clustering, including demographics, comorbidities, trigger subscale scores, and SDOH. *P* values were calculated, as appropriate, by Kruskal-Wallis rank sum test or Pearson chi-square test.

**Fig 2:**
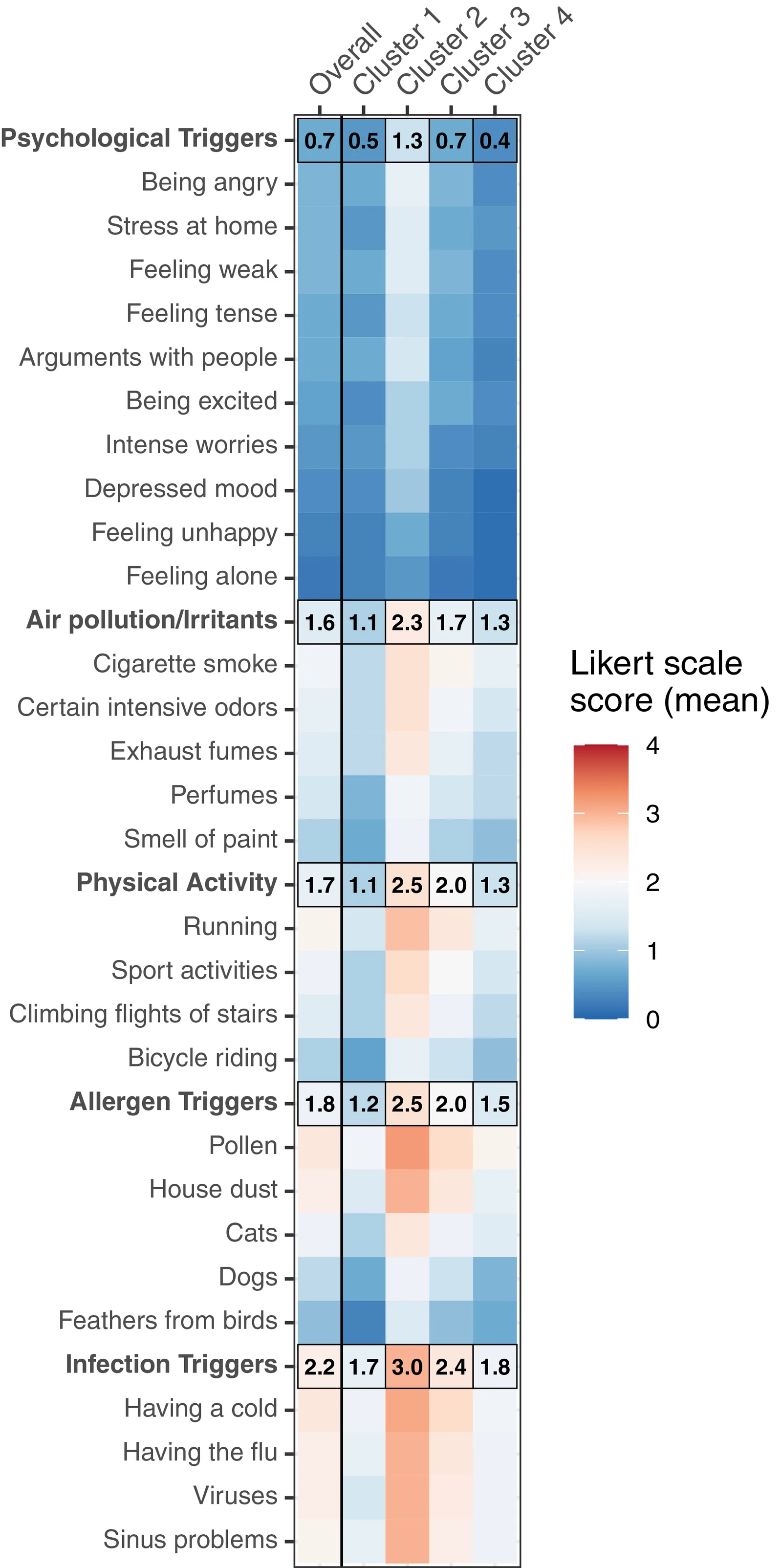
Heat map of self-reported individual triggers and trigger subscale scores across clusters. Shown are overall and within-cluster mean Likert scale scores for 5 trigger subscales *(bold)* and individual triggers making up each subscale. Individual trigger scores were derived from survey questions asking participants how often each trigger made their asthma symptoms worse with 5-point Likert scale (0-4: never, rarely, sometimes, most of the time, always). Trigger subscale scores were computed by averaging nonmissing trigger items within 5 subgroups, which were determined by PCA.

The 4 clusters differed (*P* < 10^−4^) according to trigger subscale scores, with cluster 2 having the highest within-cluster mean scores for all triggers. Fig 2, which compares the between-cluster distributions of trigger subscale scores and of the individual trigger variables within each subscale, shows that individual trigger variables followed a similar pattern to that of the trigger subscale scores. For cluster 2, *Running, Sport activities,* and *Climbing flights of stairs* contributed most to a higher Physical Activity score; exposure to *Pollen* and *House dust* contributed most to having a higher Allergen Trigger score; and *Having a cold, Having the flu, Viruses,* and *Sinus problems* contributed to a higher Infection Trigger score. Cluster 3 had higher trigger subscale scores than clusters 1 and 4, with the highest score in the Infection Trigger subscale.

All SDOH variables differed across clusters (all *P* < 10^−4^). Participants in clusters 1 and 2 reported the most social vulnerability of all clusters, with greater proportions of Medicaid health insurance (53% and 58%, respectively), high school or below education (53% and 41%, respectively), and food insecurity (84% and 87%, respectively). Participants in clusters 3 and 4 generally reported less social vulnerability, with greater proportions of private health insurance (20% and 2.7%, respectively) and lesser proportions of high school or below education (17% and 6.7%, respectively) and food insecurity (4.4% and 7.8%, respectively). Clusters 3 and 4 differed according to Medicaid health insurance (20% vs 2.7%, respectively) having an advanced degree (7.9% vs 49%, respectively), and exposure to violence (17% vs 6.2%, respectively).

### Differences in asthma history, medication receipt, and care-related behavior across clusters

Clusters differed significantly in terms of asthma history, medication receipt, and care-related behavior variables, which were not included in the clustering computation (Table V). The proportion of participants reporting an ED visit for asthma in the last year was highest in clusters 1 and 2 (both 42%), followed by cluster 3 (24%) and cluster 4 (6.2%) (*P* < 10^−4^). Similar patterns were observed for overnight hospital stays (15% cluster 1, 21% cluster 2) and 10+ days missed of work or school as a result of asthma (15% cluster 1, 21% cluster 2). Participants in cluster 4 had a later age at asthma onset (mean [IQR], 25 [10-40] years) compared to participants in clusters 1, 2, and 3. Compared to clusters 3 and 4, patients in clusters 1 and 2 were also significantly more likely to seek asthma care in the emergency room, more likely to discuss triggers with health care providers at the emergency room, and more likely to obtain asthma medications from ED prescriptions or borrow from family or friends (*P* < 10^−4^) (see Table E6 in the Online Repository available at www.jaci-global.org). Additional results can be found in the Results section in the Online Repository.

**Table V:** Participant asthma history and medication receipt by cluster

Characteristic	Overall (N = 974)	Cluster				*P*
		1 (n = 73)	2 (n = 224)	3 (n = 227)	4 (n = 450)	
**Asthma history**						
Age (years) at asthma onset	16 (5, 32)	9 (3, 24)	11 (4, 22)	11 (4, 25)	25 (10, 40)	<10^−4^
ED visits (past year)						<10^−4^
No	737 (76%)	36 (49%)	120 (54%)	168 (74%)	413 (92%)	
Yes	206 (21%)	31 (42%)	93 (42%)	54 (24%)	28 (6.2%)	
Unknown	31 (3.2%)	6 (8.2%)	11 (4.9%)	5 (2.2%)	9 (2.0%)	
Overnight hospital stays (past year)						<10^−4^
No	865 (89%)	60 (82%)	168 (75%)	207 (91%)	430 (96%)	
Yes	89 (9.1%)	11 (15%)	48 (21%)	16 (7.0%)	14 (3.1%)	
Unknown	20 (2.1%)	2 (2.7%)	8 (3.6%)	4 (1.8%)	6 (1.3%)	
ICU visits (past year)						2.8 × 10^−4^
No	926 (95%)	67 (92%)	202 (90%)	218 (96%)	439 (98%)	
Yes	28 (2.9%)	5 (6.8%)	15 (6.7%)	4 (1.8%)	4 (0.9%)	
Unknown	20 (2.1%)	1 (1.4%)	7 (3.1%)	5 (2.2%)	7 (1.6%)	
Intubations (past year)						.035
No	946 (97%)	72 (99%)	210 (94%)	225 (99%)	439 (98%)	
Yes	4 (0.4%)	0	2 (0.9%)	0	2 (0.4%)	
Unknown	24 (2.5%)	1 (1.4%)	12 (5.4%)	2 (0.9%)	9 (2.0%)	
Work/school days missed (past year)						<10^−4^
0	644 (66%)	40 (55%)	105 (47%)	140 (62%)	359 (80%)	
1-9	164 (17%)	15 (21%)	52 (23%)	48 (21%)	49 (11%)	
10+	121 (12%)	11 (15%)	48 (21%)	31 (14%)	31 (6.9%)	
Unknown	45 (4.6%)	7 (9.6%)	19 (8.5%)	8 (3.5%)	11 (2.4%)	
**Asthma medication**						
SABA						.0045
No	59 (6.1%)	2 (2.7%)	7 (3.1%)	8 (3.5%)	42 (9.3%)	
Yes	907 (93%)	70 (96%)	214 (96%)	219 (96%)	404 (90%)	
Unknown	8 (0.8%)	1 (1.4%)	3 (1.3%)	0	4 (0.9%)	
Inhaled corticosteroid						.011
No	228 (23%)	16 (22%)	40 (18%)	54 (24%)	118 (26%)	
Yes	692 (71%)	48 (66%)	174 (78%)	166 (73%)	304 (68%)	
Unknown	54 (5.5%)	9 (12%)	10 (4.5%)	7 (3.1%)	28 (6.2%)	
Prednisone						3.0 × 10^−4^
No	854 (88%)	54 (74%)	189 (84%)	198 (87%)	413 (92%)	
Yes	46 (4.7%)	9 (12%)	14 (6.3%)	13 (5.7%)	10 (2.2%)	
Unknown	74 (7.6%)	10 (14%)	21 (9.4%)	16 (7.0%)	27 (6.0%)	
Biologics						.0020
No	815 (84%)	55 (75%)	171 (76%)	195 (86%)	394 (88%)	
Yes	73 (7.5%)	10 (14%)	21 (9.4%)	16 (7.0%)	26 (5.8%)	
Unknown	86 (8.8%)	8 (11%)	32 (14%)	16 (7.0%)	30 (6.7%)	

Shown are no. (%) of participants in each level of all categorical variables, and median (IQR) for discrete variables for participants across clusters. Characteristics shown correspond to variables derived from participants’ survey responses but not used as inputs in hierarchical clustering, including asthma history and receipt of asthma medication. *P* values were calculated, as appropriate, by Kruskal-Wallis rank sum test or Pearson chi-square test.

*ICU,* Intensive care unit; *SABA,* short-acting β_2_-agonist.

## Discussion

We analyzed survey data for 974 adults with asthma who were demographically representative of Penn Medicine’s large and diverse asthma patient population. Our multivariable regression and unsupervised clustering approach allowed for combined assessment of patient-reported asthma trigger burden, which remains a relatively understudied predictor of clinical outcomes, and SDOH, which interact to shape risk of adverse asthma outcomes. Taken together, our results demonstrate the value of collecting self-reported patient data and applying clustering methods to identify distinct patient groups, enabling complex population-level assessments that move beyond reliance on single or limited variables. Future work can identify a subset of SDOH factors and asthma triggers that could be integrated into EHR patient questionnaires, enabling personalized, patient-centered asthma management.

Our univariable ZIP regression models showed that several demographic characteristics and indicators of social vulnerability increased proportionally with asthma ED visits, a finding that is now well established in observational studies. ED visits were positively associated with housing, food, transportation, energy, and communication insecurity as well as exposure to violence, and negatively associated with holding a bachelor’s or advanced degree. The US Centers for Disease Control and Prevention Asthma Call-Back Survey has similarly showed that low annual household income, low educational attainment, and cost barriers to medical care were associated with worse asthma control.^38^ Compared to SDOH, the associations between patient or physician-reported trigger burden and asthma outcomes are relatively understudied. A 2016 study by Ritz et al^39^ reported associations between asthma exacerbations and patient-reported asthma triggers, finding the strongest associations for psychological and infection triggers. This finding was replicated in our cohort. Interestingly, we also observed that although psychological triggers were strongly associated with a higher rate of ED visits (Table II), participants reported fewer psychological triggers compared to any other trigger type (Table I).

In multivariable ZIP regression analysis, we observed marked attenuation of the effects of SDOH and asthma trigger variables, while Black race remained strongly associated with ED visits. Although the absence of inflated standard errors suggested that all variables could feasibly be included in the same model, we reasoned that the effect attenuation was partly attributable to weak to moderate correlations among these variables (Fig E2) and proceeded to further investigate variable relationships via a hierarchical multiple regression analysis. The strong attenuation of ED visit IRRs for all trigger subscales in the SDOH-adjusted versus SDOH-unadjusted ZIP regression models suggests that SDOH partially explained the relationship between asthma triggers and ED visits (Table III); explicitly including race in the models yielded similar results (Table E5), suggesting that the dominant effect of race in the multivariable model was due to variables not captured by the survey.

To further explicate the relationships between asthma triggers and SDOH that were driving the attenuation observed in the multivariable model, we modeled each trigger subscale as an individual outcome in separate linear regression models. We found that psychological triggers were associated with Medicaid health insurance, lower educational attainment, and transportation insecurity; air pollution/irritant triggers were associated with food insecurity; and physical activity triggers were associated with food insecurity (*P* < .01) (Table E4). Allergens and infection triggers were not associated with any SDOH. Past studies have reported links between individual asthma triggers and SDOH, including associations between substandard housing and exposure to pests and other allergens,^20^ between overcrowding and susceptibility to upper respiratory infections,^40^ and between living in poverty and chronic psychosocial stress.^41^ Evidently social factors and asthma triggers are closely intertwined—for example, patients’ home environments are a major determinant of trigger exposures, and financial security determines one’s ability to make home modifications that mitigate triggers. However, to our knowledge, there are few studies that simultaneously model self-reported asthma trigger burden, SDOH, and asthma outcomes in a single analytical framework. Future work is needed to further explicate and clarify these relationships, such as determining whether SDOH act as a mediator or confounder of the relationship between asthma triggers and adverse outcomes.

Our clustering analysis allowed for the identification of patterns in the underlying data that resulted in interesting groupings with respect to demographics, SDOH burden, asthma trigger burden, and asthma outcomes. The goals of our cluster analysis were similar to past efforts to phenotype asthma in that we aimed to identify groups of patients with different asthma management needs,^42^^,^^43^ but different in that groups we identified were differentiated primarily by modifiable risk factors. With respect to demographic characteristics, cluster 1 had the highest proportion of male participants and cluster 4 had the highest proportion of White participants. Clusters 1 and 2 had the greatest social vulnerability, cluster 3 had moderate social vulnerability, and cluster 4 had the least social vulnerability (Table IV). Cluster 2 had the greatest trigger burden, followed by cluster 3. Taken together with patterns of asthma outcomes and health care utilization behaviors, striking racial disparities became apparent. Clusters 1 and 2 not only contained the most socially vulnerable participants and the greatest proportion of participants with ED visits, overnight hospital stays, and intensive care unit visits due to asthma but also had the highest proportions of participants of Black race (Tables IV and V). Importantly, cluster 3 had a higher trigger burden than cluster 1, yet less social vulnerability and fewer adverse asthma outcomes. These results are consistent with national trends in the United States showing marked disparities in asthma outcomes,^26^, ^27^, ^28^^,^^31^^,^^44^ which result from many factors operating concurrently on multiple scales.^31^^,^^32^

Participants’ care-related behaviors also showed striking differences across clusters, which provides insight into possible interventions. The clusters with the most social vulnerability (ie, clusters 1 and 2) and most adverse asthma outcomes also most heavily relied on the ED for seeking asthma care and obtaining medications (Table E6), consistent with previous reports that emergency health service use is associated with health care access barriers for patients with asthma.^45^^,^^46^ This finding underscores the need for a multidisciplinary approach to asthma care that includes social need screening and social work referral. In a 2023 Work Group Report, the American Academy of Allergy, Asthma & Immunology called for such an approach as crucial for all aspects of asthma management.^32^ Cluster 2 also had the highest asthma trigger burden, suggesting a possible additional point for intervention. Despite guideline recommendations, primary care providers infrequently obtain environmental histories or provide allergen mitigation counseling.^47^ Furthermore, our findings indicate that SDOH partially mediate the relationship between asthma triggers and ED visits, suggesting that clinical interventions addressing modifiable risk factors should be implemented within a framework that acknowledges and targets underlying structural determinants. We plan future studies to formally examine the roles of home exposures, patterns of health care use, access to pulmonary and allergy specialists, and allergy testing to further understand the observed interactions and implications for asthma management practices.

This study is strengthened by the high granularity of survey items, which allowed us to understand participants’ individual asthma history and SDOH exposures in detail rather than relying on composite measures or neighborhood-level indexes. The demographic representativeness of our sample with respect to Penn Medicine’s total patient population increases the likelihood that our results are generalizable to the regional population of adults with asthma, although generalizability may be limited outside of the Penn Medicine catchment area. Our study is subject to limitations, including survey-related recall bias, self-selection bias, and missingness. Because we recruited via email and administered the survey online, participants without access to an electronic device may have been unable to participate. Furthermore, unmeasured confounders may have biased our results, including nonfinancial barriers to preventative therapy (eg, underutilization of asthma controller medications, providers’ and patients’ perceptions of disease severity) and genetic risk factors.^27^

In conclusion, we surveyed adults with asthma from a large health system and found that concurrent assessment of patient-reported SDOH and asthma trigger data demonstrated utility in identifying high-risk subpopulations requiring targeted interventions such as trigger-focused counseling and multidisciplinary care that centers patients’ social needs. These findings present a framework for future work to identify the subset of SDOH factors and asthma triggers that could be captured via patient questionnaires for inclusion in EHRs to improve personalized care and address asthma disparities.

**Key messages**

• Combined assessment of patient-reported asthma triggers and SDOH using clustering methods identified distinct patient subgroups, enabling multifactorial risk stratification that moves beyond single-variable risk assessment.
• Clusters of people with asthma who reported more social vulnerability also reported more asthma-related ED visits, hospitalizations, and days missed of work or school.
• SDOH variables partially attenuated asthma trigger-outcome associations, indicating that clinical interventions should address both modifiable risk factors and underlying structural determinants.

## Disclosure statement

Supported by the National Institutes of Health (NIH) National Heart, Lung, and Blood Institute (www.nhlbi.nih.gov) under awards R01HL162354, R01HL143364, and R03HL171424; and by the National Institute of Environmental Health Sciences (www.niehs.nih.gov) under award P30ES013508. B. E. Himes contributed to this article as an employee of the University of Pennsylvania. The funders had no role in study design, data collection and analysis, decision to publish, or preparation of the report. The content is solely the responsibility of the authors and does not necessarily represent the official views of the NIH or other funding agencies.

Disclosure of potential conflict of interest: The authors declare that they have no relevant conflicts of interest.

## Supplementary data

Supplementary Figure E1

Supplementary Figure E2

Supplementary Figure E3

Supplementary Material 3

Supplementary Tables E1-E6
