# Supplementary material for: Clustering of self-reported social and environmental risk factors identifies vulnerable subgroups with increased asthma morbidity
Source: J Allergy Clin Immunol Glob. 2026 Aug 14;5(6):100775. doi: 10.1016/j.jacig.2026.100775 (PMC13545429; doi:10.1016/j.jacig.2026.100775)
Supplement: Supplementary file 1 [file mmc1.pdf]

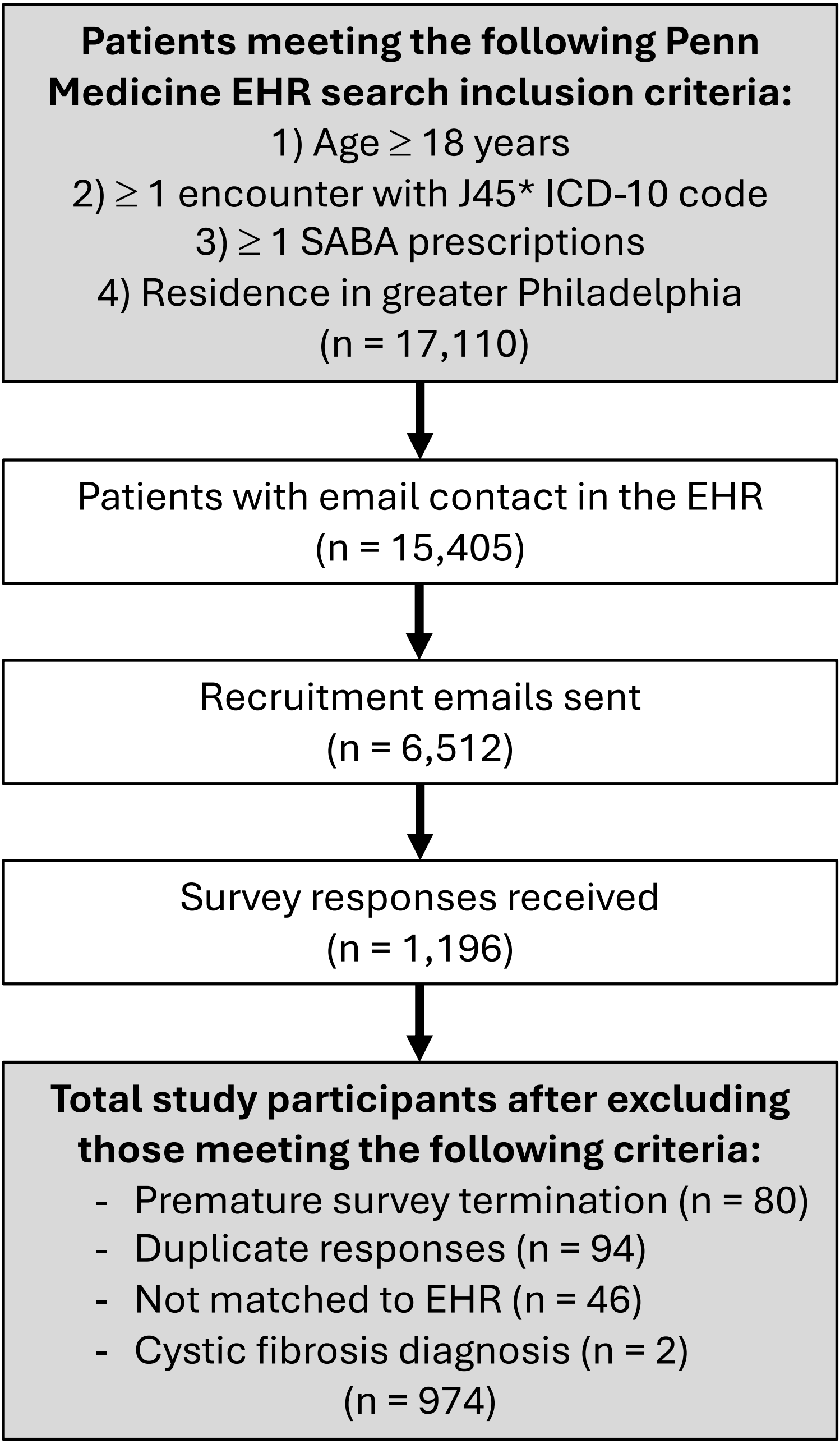
Patients meeting the following Penn Medicine EHR search inclusion criteria:
1) Age ≥ 18 years
2) ≥ 1 encounter with J45* ICD-10 code
3) ≥ 1 SABA prescriptions
4) Residence in greater Philadelphia
(n = 17,110)
Patients with email contact in the EHR
(n = 15,405)
Recruitment emails sent
(n = 6,512)
Survey responses received
(n = 1,196)
Total study participants after excluding those meeting the following criteria:
- Premature survey termination (n = 80)
- Duplicate responses (n = 94)
- Not matched to EHR (n = 46)
- Cystic fibrosis diagnosis (n = 2)
(n = 974)
