# Supplementary material for: Clustering of self-reported social and environmental risk factors identifies vulnerable subgroups with increased asthma morbidity
Source: J Allergy Clin Immunol Glob. 2026 Aug 14;5(6):100775. doi: 10.1016/j.jacig.2026.100775 (PMC13545429; doi:10.1016/j.jacig.2026.100775)
Supplement: Supplementary file 2 [file mmc2.pdf]

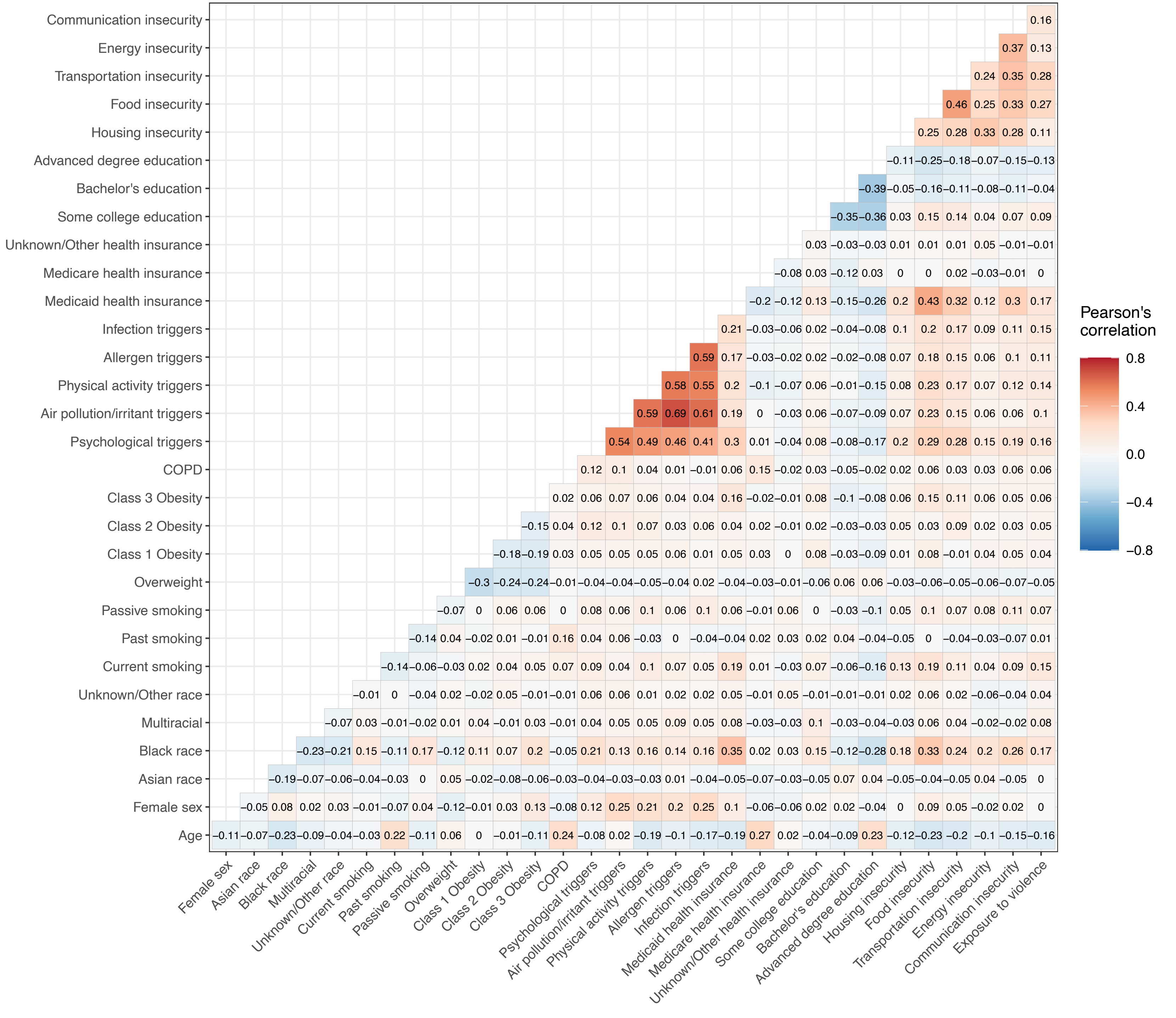
Communication insecurity 0.16
Energy insecurity 0.37 0.13
Transportation insecurity 0.24 0.35 0.28
Food insecurity 0.46 0.25 0.33 0.27
Housing insecurity 0.25 0.28 0.33 0.28 0.11
Advanced degree education −0.11 −0.25 −0.18 −0.07 −0.15 −0.13
Bachelor's education −0.39 −0.05 −0.16 −0.11 −0.08 −0.11 −0.04
Some college education −0.35 −0.36 0.03 0.15 0.14 0.04 0.07 0.09
Unknown/Other health insurance 0.03 −0.03 −0.03 0.01 0.01 0.01 0.05 −0.01 −0.01
Medicare health insurance −0.08 0.03 −0.12 0.03 0 0 0.02 −0.03 −0.01 0
Medicaid health insurance −0.2 −0.12 0.13 −0.15 −0.26 0.2 0.43 0.32 0.12 0.3 0.17
Infection triggers 0.21 −0.03 −0.06 0.02 −0.04 −0.08 0.1 0.2 0.17 0.09 0.11 0.15
Allergen triggers 0.59 0.17 −0.03 −0.02 0.02 −0.02 −0.08 0.07 0.18 0.15 0.06 0.1 0.11
Physical activity triggers 0.58 0.55 0.2 −0.1 −0.07 0.06 −0.01 −0.15 0.08 0.23 0.17 0.07 0.12 0.14
Air pollution/irritant triggers 0.59 0.69 0.61 0.19 0 −0.03 0.06 −0.07 −0.09 0.07 0.23 0.15 0.06 0.06 0.1
Psychological triggers 0.54 0.49 0.46 0.41 0.3 0.01 −0.04 0.08 −0.08 −0.17 0.2 0.29 0.28 0.15 0.19 0.16
COPD 0.12 0.1 0.04 0.01 −0.01 0.06 0.15 −0.02 0.03 −0.05 −0.02 0.02 0.06 0.03 0.03 0.06 0.06
Class 3 Obesity 0.02 0.06 0.07 0.06 0.04 0.04 0.16 −0.02 −0.01 0.08 −0.1 −0.08 0.06 0.15 0.11 0.06 0.05 0.06
Class 2 Obesity −0.15 0.04 0.12 0.1 0.07 0.03 0.06 0.04 0.02 −0.01 0.02 −0.03 −0.03 0.05 0.03 0.09 0.02 0.03 0.05
Class 1 Obesity −0.18 −0.19 0.03 0.05 0.05 0.05 0.06 0.01 0.05 0.03 0 0.08 −0.03 −0.09 0.01 0.08 −0.01 0.04 0.05 0.04
Overweight −0.3 −0.24 −0.24 −0.01 −0.04 −0.04 −0.05 −0.04 0.02 −0.04 −0.03 −0.01 −0.06 0.06 0.06 −0.03 −0.06 −0.05 −0.06 −0.07 −0.05
Passive smoking −0.07 0 0.06 0.06 0 0.08 0.06 0.1 0.06 0.1 0.06 −0.01 0.06 0 −0.03 −0.1 0.05 0.1 0.07 0.08 0.11 0.07
Past smoking −0.14 0.04 −0.02 0.01 −0.01 0.16 0.04 0.06 −0.03 0 −0.04 −0.04 0.02 0.03 0.02 0.04 −0.04 −0.05 0 −0.04 −0.03 −0.07 0.01
Current smoking −0.14 −0.06 −0.03 0.02 0.04 0.05 0.07 0.09 0.04 0.1 0.07 0.05 0.19 0.01 −0.03 0.07 −0.06 −0.16 0.13 0.19 0.11 0.04 0.09 0.15
Unknown/Other race −0.01 0 −0.04 0.02 −0.02 0.05 −0.01 −0.01 0.06 0.06 0.01 0.02 0.02 0.05 −0.01 0.05 −0.01 −0.01 −0.01 0.02 0.06 0.02 −0.06 −0.04 0.04
Multiracial −0.07 0.03 −0.01 −0.02 0.01 0.04 −0.01 0.03 −0.01 0.04 0.05 0.05 0.09 0.05 0.08 −0.03 −0.03 0.1 −0.03 −0.04 −0.03 0.06 0.04 −0.02 −0.02 0.08
Black race −0.23 −0.21 0.15 −0.11 0.17 −0.12 0.11 0.07 0.2 −0.05 0.21 0.13 0.16 0.14 0.16 0.35 0.02 0.03 0.15 −0.12 −0.28 0.18 0.33 0.24 0.2 0.26 0.17
Asian race −0.19 −0.07 −0.06 −0.04 −0.03 0 0.05 −0.02 −0.08 −0.06 −0.03 −0.04 −0.03 −0.03 0.01 −0.04 −0.05 −0.07 −0.03 −0.05 0.07 0.04 −0.05 −0.04 −0.05 0.04 −0.05 0
Female sex −0.05 0.08 0.02 0.03 −0.01 −0.07 0.04 −0.12 −0.01 0.03 0.13 −0.08 0.12 0.25 0.21 0.2 0.25 0.1 −0.06 −0.06 0.02 0.02 −0.04 0 0.09 0.05 −0.02 0.02 0
Age −0.11 −0.07 −0.23 −0.09 −0.04 −0.03 0.22 −0.11 0.06 0 −0.01 −0.11 0.24 −0.08 0.02 −0.19 −0.1 −0.17 −0.19 0.27 0.02 −0.04 −0.09 0.23 −0.12 −0.23 −0.2 −0.1 −0.15 −0.16
Female sex
Asian race
Black race
Multiracial
Unknown/Other race
Current smoking
Past smoking
Passive smoking
Overweight
Class 1 Obesity
Class 2 Obesity
Class 3 Obesity
COPD
Psychological triggers
Air pollution/irritant triggers
Physical activity triggers
Allergen triggers
Infection triggers
Medicaid health insurance
Medicare health insurance
Unknown/Other health insurance
Some college education
Bachelor's education
Advanced degree education
Housing insecurity
Food insecurity
Transportation insecurity
Energy insecurity
Communication insecurity
Exposure to violence
Pearson's correlation
0.8
0.4
0.0
−0.4
−0.8
