# Supplementary material for: Clustering of self-reported social and environmental risk factors identifies vulnerable subgroups with increased asthma morbidity
Source: J Allergy Clin Immunol Glob. 2026 Aug 14;5(6):100775. doi: 10.1016/j.jacig.2026.100775 (PMC13545429; doi:10.1016/j.jacig.2026.100775)
Supplement: Supplementary file 3 [file mmc3.pdf]

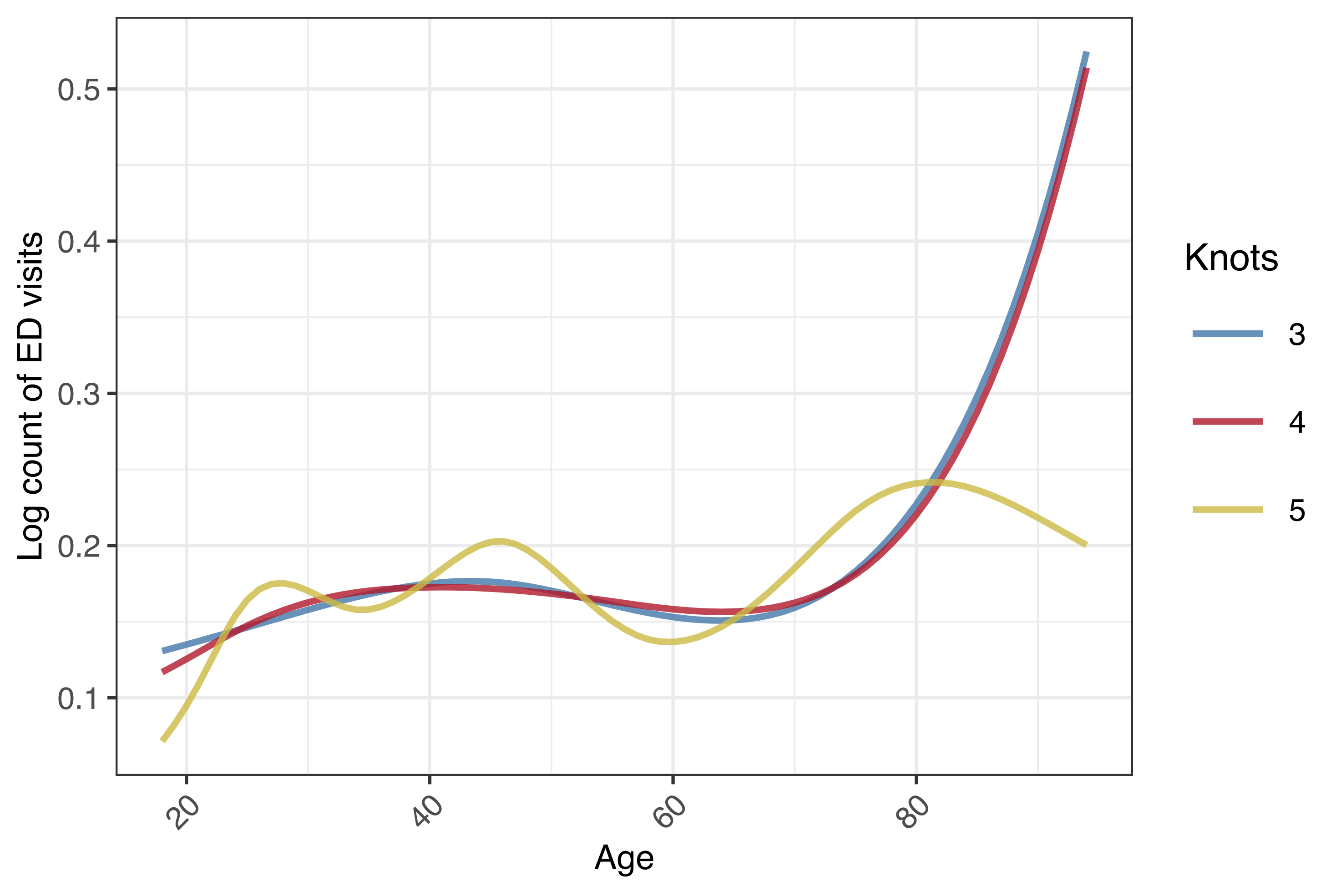

Log count of ED visits
0.1
0.2
0.3
0.4
0.5
20
40
60
80
Age
Knots
3
4
5
