# Supplementary material for: Clustering of self-reported social and environmental risk factors identifies vulnerable subgroups with increased asthma morbidity
Source: J Allergy Clin Immunol Glob. 2026 Aug 14;5(6):100775. doi: 10.1016/j.jacig.2026.100775 (PMC13545429; doi:10.1016/j.jacig.2026.100775)
Supplement: Supplementary file 4 [file mmc4.docx]

**S1 Text. Supplemental Methods**

**Survey design**

As part of the *Penn Airways Disease EHR-Based Study (PADES)*, we developed a 20–30-minute survey to collect patient-reported information aimed at identifying factors associated with asthma exacerbations and guiding the design of effective regional and personalized interventions to reduce asthma symptoms among Penn Medicine patients. Demographic questions to capture gender and race/ethnicity were adapted from those available in PhenX Toolkit under IDs 011801 and 011901, respectively (1). Asthma history, asthma medication use, smoking history, BMI, and SDOH questions were adapted from a clinical trial protocol used in a study of Philadelphia area asthma patients (2). This included validated food insecurity screening questions from the Hunger Vital Sign (3). Asthma trigger questions were adapted from the Asthma Trigger Inventory (4).

**Survey measures**

The full survey questionnaire is available in S2 Text.

*Demographic and asthma comorbidity variables*

Demographic variables included participants’ age, sex, race, and ethnicity. Health factors which have been associated with asthma included smoking history, body mass index (BMI), and comorbid chronic obstructive pulmonary disease (COPD).

*Asthma history and medications*

Variables related to asthma exacerbations were measured using survey questions assessing self-reported asthma-related emergency department (ED) visits, overnight hospital stays, ICU visits, intubations, and days missed of work or school over the last 12 months. A series of survey questions asked participants to indicate which classes of asthma medications they currently take. Participants were provided with generic and brand-names along with pictures to aid in classifying their medications.

*SDOH measures*

SDOH were captured by asking participants whether over the last 12 months they had experienced neighborhood violence, housing insecurity, food insecurity, transportation insecurity, energy insecurity (i.e. lack of access to electricity/gas), or communication insecurity (i.e. lack of access to telephone/cellphone service). Participants were also asked questions about their health insurance type and educational attainment.

*Asthma triggers*

Asthma triggers were assessed by providing a list of 28 common triggers and asking participants to indicate how often each trigger made their asthma symptoms worse using a 5-point Likert scale (0-4; *Never, Rarely, Sometimes, Most of the time, Always*) with an additional *I’m not sure* option.

*Access to asthma care*

Several survey questions assessed participants’ access to asthma care. Participants were asked to report where they usually go to seek care for asthma symptoms (i.e. primary care, urgent care, etc.) and how they obtain asthma medications (i.e. prescriptions from a doctor, free samples, borrowing, etc.). Participants were also asked to indicate how, if at all, they discuss their asthma triggers with healthcare providers (i.e. at annual visits, in the ED, etc.).

**Inclusion and exclusion criteria for invitation to complete survey**

EHR data corresponding to Penn Medicine encounters between January 2017 and March 2023 containing at least one asthma ICD-10 code (i.e., J45) was used to identify patients eligible to receive an invitation to complete the survey according to the following inclusion criteria: 1) at least 18 years of age at first encounter, 2) at least one instance of a *primary* asthma diagnosis based on ICD-10 of J45* in an inpatient, outpatient, or emergency department visit, 3) at least one prescription for an inhalation-route short-acting β_2_-agonist (SABA), 4) residence in the greater Philadelphia area (i.e., Pennsylvania counties: Delaware, Chester, Montgomery, Bucks, Philadelphia or New Jersey counties: Camden, Gloucester, Burlington), and 5) email address available in the EHR. Patients with an indication of expiration in the EHR were excluded.

**Survey administration**

The survey was deployed electronically via Penn REDCap from June 2023 to May 2024. Eligible participants were asked to provide electronic consent, and those who did so could begin answering questions. Participants who completed the survey received a $30 gift card as payment. Surveys were deployed to eligible participants in batches every two weeks, with two reminders per batch occurring one week apart, until at least 1000 responses were collected. Eligible participants were selected at random initially, but later at random within race/ethnicity and sex strata, such that the cohort recruited would have sex, race, and ethnicity distributions matching those of the underlying Penn Medicine asthma patient population according to EHR data used to recruit participants and our prior studies using Penn Medicine EHR data (i.e., approximately 70% female, 39% Black, 48% White, 12% Other race groups, 5% Hispanic) (5–7). In addition, participants from Other race groups were recruited in proportions higher than their underlying distribution to try to ensure that all groups were adequately represented for statistical analyses.

**Inclusion and exclusion criteria for selection of study participants**

Following survey administration, additional criteria were applied to select participants eligible for inclusion in the reported analysis. Participants with incomplete or duplicate survey responses were excluded from analysis, as were participants whose biographic information could not be matched to the list of eligible participants based on EHR data. Participants who self-reported a cystic fibrosis diagnosis in their survey responses were also excluded.

**Definitions of variables used in statistical analyses**

*Demographic and asthma comorbidity variables*

Demographic variables were obtained from EHR data or survey responses, cleaned, and included as independent variables in our analysis. Participants’ age (recorded as an integer in years) and sex were obtained from the codified EHR data using the most recent entry in the EHR at the time the dataset was extracted (March 2023). Age was categorized based on multivariable spline analysis (see *Spline analysis* below). Race and ethnicity were captured from survey questions and classified as *American Indian or Alaskan Native, Asian, Black, Hispanic or Latino, White, Middle Eastern or North African, Native Hawaiian or Pacific Islander,* or *Multiracial,* where *Multiracial* contained participants who selected more than one race or ethnicity. Smoking history was captured from survey questions as *None, Current, Past,* or *Passive*, where *Passive* contained participants who reported exposure to smoke at work or in their household. Body mass index (BMI) and chronic obstructive pulmonary disease (COPD) status were captured from survey questions. BMI was categorized using standard CDC categories as: *Not overweight or obese* (<25.0 kg/m^2^), *Overweight* (25.0 to <30.0 kg/m^2^), *Class 1 obesity* (30.0 to <35.0 kg/m^2^), *Class 2 obesity* (35.0 to <40.0 kg/m^2^), and *Class 3 obesity* (40.0 kg/m^2^). All variables included either an *Unknown* level, which captured nonrespondents, or an *Unknown/Other* level, which captured nonrespondents and response levels with insufficient observations to yield meaningful statistical insights. For variables in which the *Unknown* level itself had substantially inflated standard errors in statistical models due to insufficient observations (smoking history: n = 12), this level was dropped from regression analyses.

*Asthma history and medications*

Self-reported asthma-related ED visits in the last year (ED visits) was the primary outcome used for statistical analyses. We fit ZIP models with ED visits as a count outcome with levels *0, 1, 2, 3, 4+*, where all counts ≥ 4 were aggregated as 4+. Participants who did not respond to the ED visit question in the survey (n = 31) were excluded from analyses in which ED visits was the outcome.

Several additional variables captured asthma history and medications but were not included in regression analysis. These variables characterized self-reported asthma history measures, including overnight hospital stays, ICU visits, and intubations (captured as *Yes/No* indicators), as well as days missed of work or school over the last 12 months (captured as *0, 1-9, 10+).* Additional variables capturing asthma medication use included *SABA, ICS, prednisone,* and *biologics*, captured as *Yes/No* indicators. All variables included *Unknown* levels.

*SDOH measures*

Variables were created from survey questions that asked participants about several SDOH-related issues over the last 12 months. These variables included exposure to neighborhood violence, housing insecurity, food insecurity, transportation insecurity, energy insecurity (i.e. lack of access to electricity/gas), and communication insecurity (i.e. lack of access to telephone/cellphone service), all of which were captured as *Yes/No* indicators. Additional SDOH variables included health insurance type (captured as *Private*, *Medicaid, Medicare, Military,* or *None*) and educational attainment (captured as *High school or below, Some college, Bachelor, or Advanced degree*), previously used proxies for socioeconomic status (5,8)*.* The health insurance type and violence survey questions included an *Other* response option for which we reviewed responses and recategorized them into the appropriate category*.* All variables included either an *Unknown* level, which captured nonrespondents, or an *Unknown/Other* level, which captured nonrespondents and response levels with insufficient observations. For variables in which the *Unknown* level had substantially inflated standard errors in statistical models due to insufficient observations (education: n = 8; food insecurity: n = 2; transportation insecurity: n = 7), this level was dropped from regression analyses.

*Care-related behaviors*

Individual survey variables that sought to characterize issues related to asthma management included: Primary method of seeking asthma care (levels: *primary care office, specialist office, urgent care, emergency room, other*), Method of discussing triggers with healthcare team (multiple choice levels: *annual visits, phone, emergency room visits, never*) and Method of obtaining asthma medications (multiple choice levels: *doctor prescription, ED prescription, doctor sample, ED sample, borrowing, other*). These variables were not included in regression analyses.

**Statistical analysis**

*Asthma trigger subscale scores*

PCA solutions with between four and eight factors were explored to reduce the dimensionality of the 28 trigger variables into subscales. Trigger subscale score variables were then computed by averaging non-missing trigger items within each factor excluding nonresponses and *I’m not sure* responses. As a result, within each subscale, participants who responded only *I’m not sure* or did not respond to any trigger questions were coded as missing. These participants (psychological triggers: n = 73; air pollutant/irritant triggers: n = 52; physical activity triggers: n = 45; allergen triggers: n = 31; infection triggers: n = 29) were excluded from regression analyses.

*Spline analysis of the relationship between ED visits and age*

To support model interpretability while appropriately representing the potential nonlinear relationship between age and ED visits for asthma, multivariable ZIP regression models were fit with age modeled as a natural cubic spline. The models were evaluated on modified datasets which varied age while holding all other independent variables constant at their reference level. Knot locations were based on Harrell’s recommended percentiles (9). We then examined how the log count of ED visits varied over the distribution of the age variable to guide categorization of age into meaningful groups.

*Hierarchical multiple regression models*

Several *post hoc* analyses were run to better understand the relationships among trigger subscale scores, SDOH, and ED visits. Two-step hierarchical ZIP regression models were fit with ED visits as the outcome while adjusting for each trigger subscale one at a time. For each of these models, age, sex, and BMI were included as covariates in step 1, and the eight SDOH variables were added to each model in step 2. As a sensitivity analysis, we repeated the regression analyses with race included as an additional covariate in step 1. Additionally, linear regression models were fit to further explore associations between each trigger subscale score as a continuous outcome while including all eight SDOH variables, age, sex, and BMI as independent variables. We chose to include only a subset of demographic variables (i.e. age, sex, and BMI) as covariates in these *post hoc* analyses to focus on the relationships between trigger subscale scores and SDOH variables.

*Hierarchical agglomerative clustering*

As an alternative analysis to identify distinct subgroups of patients with asthma based on their demographic characteristics, asthma triggers, and SDOH responses, Ward's minimum-variance method with an agglomerative approach was used to perform hierarchical clustering of participants by minimizing the within-cluster error sum of squares (ESS). Variables included in the clustering were the independent variables used in the ZIP regression. Pairwise dissimilarity between participants was determined by computing the Gower distance (10), a similarity coefficient that handles mixed data types, based on the variables used for clustering. Silhouette scores were computed to determine the optimal number of clusters. Chi-squared and Kruskal-Wallis rank sum tests were used to assess the between-cluster distribution of included variables, as well as the between-cluster distribution of held-out variables related to asthma history, medication use, and care-related behaviors.

**References**

1.	Hamilton CM, Strader LC, Pratt JG, Maiese D, Hendershot T, Kwok RK, et al. The PhenX Toolkit: get the most from your measures. Am J Epidemiol. 2011 Aug 1;174(3):253–60. doi:10.1093/aje/kwr193 PubMed PMID: 21749974; PubMed Central PMCID: PMC3141081.

2.	Apter AJ, Bryant-Stephens T, Han X, Park H, Morgan A, Klusaritz H, et al. Clinic navigation and home visits to improve asthma care in low income adults with poorly controlled asthma: before and during the pandemic. Contemp Clin Trials. 2022 Jul 1;118:106808. doi:10.1016/j.cct.2022.106808

3.	Children’s HealthWatch. The Hunger Vital Sign^TM^ [Internet]. [Accessed 2026 May 29]. Available from: http://childrenshealthwatch.org/public-policy/hunger-vital-sign/

4.	Ritz T, Steptoe A, Bobb C, Harris AHS, Edwards M. The Asthma Trigger Inventory: validation of a questionnaire for perceived triggers of asthma. Biopsychosoc Sci Med. 2006 Dec;68(6):956. doi:10.1097/01.psy.0000248898.59557.74

5.	Schreibman A, Lactaoen K, Joo J, Gleeson PK, Weissman GE, Apter AJ, et al. Augmenting electronic health record data with social and environmental determinant of health measures to understand regional factors associated with asthma exacerbations. PLOS Digit Health. 2025 Jun;4(6):e0000677. doi:10.1371/journal.pdig.0000677 PubMed PMID: 40549800; PubMed Central PMCID: PMC12184914.

6.	Greenblatt RE, Zhao EJ, Henrickson SE, Apter AJ, Hubbard RA, Himes BE. Factors associated with exacerbations among adults with asthma according to electronic health record data. Asthma Res Pract. 2019;5:1. doi:10.1186/s40733-019-0048-y PubMed PMID: 30680222; PubMed Central PMCID: PMC6339400.

7.	Xie S, Greenblatt R, Levy MZ, Himes BE. Enhancing electronic health record data with geospatial information. AMIA Summits Transl Sci Proc. 2017 Jul 26;2017:123–32. PubMed PMID: 28815121; PubMed Central PMCID: PMC5543367.

8.	Redmond C, Akinoso-Imran AQ, Heaney LG, Sheikh A, Kee F, Busby J. Socioeconomic disparities in asthma health care utilization, exacerbations, and mortality: a systematic review and meta-analysis. J Allergy Clin Immunol. 2022 May 1;149(5):1617–27. doi:10.1016/j.jaci.2021.10.007 PubMed PMID: 34673047.

9.	Harrell FE. Regression modeling strategies: with applications to linear models, logistic regression, and survival analysis. 1st ed. New York, NY: Springer; 2001. (Springer Series in Statistics). doi:10.1007/978-1-4757-3462-1

10. Gower JC. A general coefficient of similarity and some of its properties. Biometrics. 1971;27(4):857–71. doi:10.2307/2528823
