# Supplementary material for: Clustering of self-reported social and environmental risk factors identifies vulnerable subgroups with increased asthma morbidity
Source: J Allergy Clin Immunol Glob. 2026 Aug 14;5(6):100775. doi: 10.1016/j.jacig.2026.100775 (PMC13545429; doi:10.1016/j.jacig.2026.100775)
Supplement: Supplementary file 5 [file mmc5.docx]

**S2 Text. Questionnaire**

**Introduction**

Allergies, pollution, and other things we encounter in the environment are known to play a role in making asthma symptoms worse for some people. However, we do not always have detailed information about these triggers for our patients in the Philadelphia region. We are trying to find out whether there are specific areas where people have worse asthma symptoms, and if so, what issues may be responsible for these trends. This information may help us design interventions to reduce asthma symptoms among our patients. Please complete the survey below to help us understand how the environment affects people with asthma.

**Consent**

First Name: __________________________________
Last Name: __________________________________
Please enter your date of birth: ______ / ______ / ______ (month / day / year)

Please enter your full current residential address (street, city, state, and zip code):

This online survey should take about 20-30 minutes to complete. Participation is voluntary, and responses will be kept confidential. If you choose not to participate, it will not affect your care or relationship with the University of Pennsylvania Health System or your provider. You can skip any question you'd rather not answer or stop the survey at any time. If you choose to complete the survey, you will receive a $30 incentive.

You may only complete the survey once.

___ I understand.

Date of Consent: __________________________________
Please enter your email: __________________________________

**General Information**

1. What terms best describe your gender identity?

SELECT ALL THAT APPLY

[ ] Woman

[ ] Man

[ ] Non-binary

[ ] Transgender

[ ] None of these describe me, and I’d like to consider additional options

Branching logic: If ‘Non-binary’, ‘Transgender,’ or ‘None of these describe me and I’d like to consider additional options’ selected:

1b. Are any of these a closer description to your gender identity?

[ ] Trans man/Transgender Man/FTM

[ ] Trans woman/Transgender Woman/MTF

[ ] Genderqueer

[ ] Genderfluid

[ ] Gender variant

[ ] Questioning or unsure of your gender identity

[ ] None of these describe me, and I want to specify

Branching logic: If ‘None of these describe me, and I want to specify’ selected: Please specify any additional categories related to gender that describe you:________________

2. Which categories describe you? Select all that apply. Note, you may select more than one group.

[ ] American Indian or Alaska Native

Branching Logic if ‘American Indian or Alaska Native’ selected:

[ ] American Indian

[ ] Alaska Native

[ ] Central or South American Indian

[ ] None of these fully describe me

▪	Branching Logic: If ‘American Indian,’ ‘Alaska Native,’ or ‘Central or South American Indian’ selected: Please provide the name of the tribe in which you are enrolled or affiliated or your tribal descent________________

▪	Branching Logic: If ‘None of these fully describe me’ selected: Please specify any additional categories related to American Indian or Alaska Native race that describe you________________

[ ] Asian

Branching Logic if ‘Asian’ selected:

[ ] Asian Indian

[ ] Cambodian

[ ] Chinese

[ ] Filipino

[ ] Hmong

[ ] Japanese

[ ] Korean

[ ] Pakistani

[ ] Vietnamese

[ ] None of these fully describe me

▪	Branching Logic: If ‘None of these fully describe me’ selected: Please specify any additional categories related to Asian race that describe you________________

[ ] Black, African American, or African

Branching Logic if ‘Black, African American, or African’ selected:

[ ] African American

[ ] Barbadian

[ ] Caribbean

[ ] Ethiopian

[ ] Ghanaian

[ ] Haitian

[ ] Jamaican

[ ] Liberian

[ ] Nigerian

[ ] Somali

[ ] South African

[ ] None of these fully describe me

▪	Branching Logic: If ‘None of these fully describe me’ selected: Please specify any additional categories related to Black, African American or African race that describe you________________

[ ] Hispanic, Latino, or Spanish

Branching Logic if ‘Hispanic, Latino, or Spanish’ selected:

[ ] Colombian

[ ] Cuban

[ ] Dominican

[ ] Ecuadorian

[ ] Honduran

[ ] Mexican or Mexican American

[ ] Puerto Rican

[ ] Salvadoran

[ ] Spanish

[ ] None of these fully describe me

▪	Branching Logic: If ‘None of these fully describe me’ selected: Please specify any additional categories related to Hispanic, Latino, or Spanish race that describe you________________

[ ] Middle Eastern or North African

Branching Logic if ‘Middle Eastern or North African’ selected:

[ ] Afghan

[ ] Algerian

[ ] Egyptian

[ ] Iranian

[ ] Iraqi

[ ] Israeli

[ ] Lebanese

[ ] Moroccan

[ ] Syrian

[ ] Tunisian

[ ] None of these fully describe me

▪	Branching Logic: If ‘None of these fully describe me’ selected: Please specify any additional categories related to Middle Eastern or North African race that describe you________________

[ ] Native Hawaiian or other Pacific Islander

Branching Logic if ‘Native Hawaiian or other Pacific Islander’ selected:

[ ] Chamorro

[ ] Chuukese

[ ] Fijian

[ ] Marshallese

[ ] Native Hawaiian

[ ] Palauan

[ ] Samoan

[ ] Tahitian

[ ] Tongan

[ ] None of these fully describe me

▪	Branching Logic: If ‘None of these fully describe me’ selected: Please specify any additional categories related to Native Hawaiian or other Pacific Islander race that describe you________________

[ ] White

Branching Logic if ‘White’ selected:

[ ] Dutch

[ ] English

[ ] European

[ ] French

[ ] German

[ ] Irish

[ ] Italian

[ ] Norwegian

[ ] Polish

[ ] Scottish

[ ] Spanish

3. What type of health insurance do you currently have?

SELECT ALL THAT APPLY

[ ] Medicaid

[ ] Medicare only

[ ] Insurance through a current or former employer or union (of yours or another family member’s). This could include COBRA coverage.

[ ] Insurance purchased directly from an insurance company (by you or another family member). This would include coverage purchased through an exchange or marketplace, such as HealthCare.gov.

[ ] TRICARE or other military health care, including VA health care.

[ ] Indian Health Service

[ ] None

[ ] Other (specify): __________________

4. How tall are you? ________________
5. How much do you weigh? ________________
6. Where do you live? (Please write your home address with zip code)

_____________________________________________

7. During the past 12 months, has there been water or dampness in your home from broken pipes, leaks, heavy rain, or floods?

- Yes
- No
- Don’t know/Not sure

8. Does your home sometimes have a mildew odor or musty smell?

- Yes, daily
- Yes, weekly
- Yes, monthly or less frequently
- No
- Don’t know/Not sure

9. Is air conditioning (refrigeration) used to cool your home?

- Yes
- No

If yes, which types of air conditioning units do you use?

SELECT ALL THAT APPLY

[ ] Central unit/units

[ ] Window or wall unit/units

[ ] Portable unit/units

[ ] Don’t know/Not sure

10. Does your household have pets such as dogs, cats, hamsters, birds or other feathered or furry pets that spend time indoors?

- Yes
- No

If yes, what kind of pet or pets does your household have?

SELECT ALL THAT APPLY

[ ] Cat

[ ] Dog

[ ] Small furry animal

[ ] Bird or other feathered animal

11. In the past 30 days, has anyone seen a cockroach inside your home?

- Yes
- No
- Don’t know/Not sure

12. In the past 30 days, has anyone seen mice or rats inside your home? Do not include mice or rats kept as pets.

- Yes
- No
- Don’t know/Not sure

13. We would like to know how you spend your time. Are you working now, looking for work, retired, keeping house, a student, or something else?

- Working now
- Only temporarily laid off, sick leave or maternity leave
- Looking for work, unemployed
- Retired
- Disabled, permanently or temporarily
- Keeping house
- Student
- Other (specify): __________________

Branching Logic: If ‘Working now’, ‘Looking for work, unemployed’, or ‘Retired’ selected: What kind of work do/did you do? (For example: registered nurse, personnel manager, supervisor of order department, secretary, accountant)

_____________________________________________

Branching Logic: If ‘Working now’, ‘Looking for work, unemployed’, or ‘Retired’ selected: What were your most important activities or duties?

_____________________________________________

Branching Logic: If ‘Working now’ selected: Regarding your current employment, do you primarily work at home or outside the home?

- Home
- Outside the home

Branching Logic: If ‘Outside the home’ selected: On an average workday, how many hours do you work outside the home?

- Less than 6 hours a day
- 6-7 hours a day
- 8-9 hours a day
- 12 or more hours a day

Branching Logic: If ‘Outside the home’ selected: At your job or business, are you exposed to any of the following?

SELECT ALL THAT APPLY

[ ] Water or dampness from broken pipes, leaks, heavy rain, or floods

[ ] Mildew odor or musty smell

[ ] Dogs

[ ] Cats

[ ] Other small furry animals, e.g. as rabbit, guinea pig or hamster

[ ] Rodents

[ ] Cockroaches

[ ] Paints or solvents, such as paint thinners and removers

[ ] Glues and adhesives, such as contact cement, super glues, and aerosol adhesives that contain chemical solvents

[ ] Pesticides

[ ] Gasoline/diesel fumes

[ ] Smoke

[ ] Dust

[ ] Cleaning solutions, including household cleaners and bleach

[ ] Perfumes, nail polishes and removers, and other cosmetic chemicals

Branching Logic: If ‘Outside the home’ selected: At this job or business, how many hours per day can you smell the smoke from other people’s cigarettes, cigars, and/or pipes?

- Never
- Less than an hour a day
- 1-2 hours a day
- 3-5 hours a day
- More than 5 hours a day

14. What is the highest grade or level of school you have completed or the highest degree you have received?

- Never attended school
- Less than 9^th^ grade
- Less than 12^th^ grade
- High school graduate or GED
- Some college, no degree
- Bachelor’s degree (BA, BS, AB, BBA)
- Master’s degree (MA, MS, MEng, MEd, MBA)
- Professional school (MD, DDS, DVM, JD)
- Doctoral degree (PhD, EdD)

15. In the past 12 months, have you ever been without housing?

- Yes
- No

16. In the past 12 months, did you worry about whether food would run out before you got money to buy more?

- Never true
- Sometimes true
- Often true

17. In the past 12 months, did the food you bought not last, and you didn’t have money to get more?

- Never true
- Sometimes true
- Often true

18. In the past 12 months, has lack of transportation kept you from medical appointments, meetings, work, or from getting things needed for daily living?

- No
- Yes, it has kept me from non-medical meetings, appointments, work, or getting things I need
- Yes, it has kept me from medical appointments or getting medications
- Yes, it has kept me from both medical and non-medical commitments

19. In the past 12 months, have you had to go without electricity or gas?

- Yes
- No

20. In the past 12 months, have you had to go without telephone or cellphone service?

- Yes
- No

21. In the past 12 months, did you witness any violence in your neighborhood?

- Yes
- No

Branching Logic: If ‘Yes’ selected: What kind of violence did you witness in your neighborhood:

SELECT ALL THAT APPLY

[ ] A fight in which a weapon was used

[ ] A violent argument between neighbors

[ ] A gang-related fight

[ ] A sexual assault or rape

[ ] A robbery or mugging

[ ] Other (specify): __________________

**Smoking History**

22. Do you currently smoke cigarettes?

- Yes
- No

23. Have you ever smoked cigarettes in the past?

- Yes
- No

Branching Logic: If ‘Yes’ selected to either question: **If yes to either question**, please answer the following questions.

1. Number of years you smoked	______
2. Average number of packs per day	______

24. Other than yourself, how many people who live or are regularly in your home smoke cigarettes, cigars, or pipes anywhere inside this home?

- No one else smokes inside the home
- 1-2 other people
- 3-4 other people
- 5 or more people

Branching Logic: If ‘1-2 other people’, ‘3-4 other people’ or ‘5 or more people’ selected: How many total cigarettes, cigars and/or pipes per day does this person (or persons) usually smoke inside the home? (Note: 1 pack equals 20 cigarettes) ______

**Asthma History**

25. How old were you when you were first diagnosed with asthma? ______ (years)
26. Has a doctor or other health professional ever told you that you have any of the conditions listed below?

Hypertension or high blood pressure	__ Yes	__ No

Diabetes	__ Yes	__ No

Hyperlipidemia or high cholesterol	__ Yes	__ No

Other heart disease	__ Yes	__ No

Cancer	__ Yes	__ No

If yes, please specify _______________________

COPD	__ Yes	__ No

Chronic bronchitis	__ Yes	__ No

Emphysema	__ Yes	__ No

Other	__ Yes	__ No

If yes, please specify _______________________

Other	__ Yes	__ No

If yes, please specify _______________________

Other	__ Yes	__ No

If yes, please specify _______________________

27. Do you use an inhaled short-acting beta-agonist, sometimes referred to as a rescue medication, such as *ProAir (Albuterol Sulfate)* or *Xopenex (Levalbuterol)* (see common ones pictured below)?

- Yes
- No
- I’m not sure

Branching Logic: If ‘Yes’ selected: How many years have you been taking a short-acting beta-agonist? ______

28. Do you use an inhaler that contains a steroid such as *Asmanex (mometasone furoate), QVar (Beclomethasone Dipropionate), Flovent (Fluticasone Propionate), Pulmicort (Budesonide), Aerobid (Flunisolide), Azmacort (Triamcinolone Acetonide), Vanceril (Beclomethasone Dipropionate), Advair (Fluticasone in combination with Salmeterol), Symbicort (Budesonide in combination with Formoterol), Arnuity Ellipta (fluticasone furoate)* (see common ones pictured below)?

- Yes
- No
- I’m not sure

Branching Logic: If ‘Yes’ selected: How many years have you been taking an inhaled steroid? ______

29. Do you take daily Prednisone for asthma?

- Yes
- No
- I’m not sure

Branching Logic: If ‘Yes’ selected: How much Prednisone (mg/day) are you currently prescribed? ______ (mg/day)

Branching Logic: If ‘Yes’ selected: How many days were you taking Prednisone in the last 6 months? ______ (days)

30. Do you take any asthma biologics such as *Xolair (Omalizumab), Nucala (Mepolizumab), Fasenra (Benralizumab), Cinqair (Reslizumab), or Dupixent (Dupilumab)?*

- Yes
- No
- I’m not sure

31. How much do you pay for your asthma medicines out of pocket on average per month? ______
32. Where do you get the majority of your asthma medications?

SELECT ALL THAT APPLY

[ ] Prescription from the doctor’s office

[ ] Prescription from the Emergency Department

[ ] Free samples at the doctor’s office

[ ] Free samples at the Emergency Department

[ ] Borrow from a friend or family member

[ ] Other, Specify __________

33. How many days of work/school have you missed for your asthma in the last 365 days? ______ *Please enter “0” if you have not missed any days for your asthma.*
34. In your lifetime, have you ever gone to the emergency room because of asthma?

- Yes
- No

Branching Logic: If ‘Yes’ selected: In the last 12 months, how many times did you go to an emergency room because of asthma? ______

Branching Logic: If ‘Yes’ selected: In your lifetime, how many times did you go to an emergency room because of asthma? ______

Branching Logic: If ‘Yes’ selected: In your lifetime, were you ever a patient overnight in a hospital because of your asthma? Don’t count overnights spent in an emergency room.

- Yes
- No

Branching Logic: If ‘Yes’ selected: In your lifetime, how many times were you a patient overnight in a hospital because of your asthma? Don’t count overnights spent in an emergency room. ______

Branching Logic: If ‘Yes’ selected: In the last 12 months, how many times were you a patient overnight in a hospital room because of your asthma? Don’t count overnights spent in an emergency room. ______

Branching Logic: If ‘Yes’ selected: In your lifetime, have you ever been admitted to an intensive care unit in a hospital because of your asthma?

- Yes
- No

Branching Logic: If ‘Yes’ selected: Altogether, how many times in your life have you been admitted to an intensive care unit in a hospital because of your asthma? ______

Branching Logic: If ‘Yes’ selected: In the last 12 months, how many times have you been admitted to an intensive care unit in a hospital because of your asthma? ______

Branching Logic: If ‘Yes’ selected: In your lifetime, have you ever been intubated for asthma?

- Yes
- No

Branching Logic: If ‘Yes’ selected: Altogether, how many times in your life have you been intubated for asthma? (Intubation is when a tube is placed down your throat to help you breath.) ______

Branching Logic: If ‘Yes’ selected: In the last 12 months, how many times in your life have you been intubated for asthma (Intubation is when a tube is placed down your throat to help you breath.) ______

35. If you were to seek care for asthma symptoms, where do you normally go?

- Urgent Care
- Emergency Room
- Primary Care Office
- I’m not sure
- Other, specify: ________

36. What hospital/ health organization(s) do you see for asthma care?

SELECT ALL THAT APPLY

[ ] Penn Medicine

[ ] Temple University

[ ] Jefferson Health

[ ] Vybe

[ ] I’m not sure

[ ] Other, specify: ________

37. Are there times of the year when your asthma symptoms are worse? If so, select the months when you have worse symptoms:

[ ] Jan [ ] Feb [ ] Mar [ ] Apr [ ] May [ ] Jun [ ] Jul [ ] Aug [ ] Sept [ ] Oct [ ] Nov [ ] Dec

**Asthma Triggers**

38. There are many different causes for asthma symptoms. Situations causing symptoms can vary considerably from one person to the other. Do you have any asthma triggers, or things that make your asthma symptoms get worse (such as *smoke, dust, gas/diesel fumes, smog, cleaning products, furry animals, mold, or outdoor pollens)*?

- Yes
- No

Branching Logic: If ‘Yes’ selected: Please indicate for each of the listed causes below how often they are involved when you experience symptoms of asthma. Please base your answers on your own personal experience, not on what you think should lead to asthma for the typical patient. The following things can trigger my asthma alone or in part: (for each trigger please circle the number that applies most to you).

|  | I’m Not Sure | Never | Rarely | Sometimes | Most of the time | Always |
| --- | --- | --- | --- | --- | --- | --- |
| Having a cold | 0 | 1 | 2 | 3 | 4 | 5 |
| Cigarette smoke | 0 | 1 | 2 | 3 | 4 | 5 |
| Running | 0 | 1 | 2 | 3 | 4 | 5 |
| Being angry | 0 | 1 | 2 | 3 | 4 | 5 |
| Pollen | 0 | 1 | 2 | 3 | 4 | 5 |
| Feeling alone | 0 | 1 | 2 | 3 | 4 | 5 |
| Exhaust fumes | 0 | 1 | 2 | 3 | 4 | 5 |
| Bicycle riding | 0 | 1 | 2 | 3 | 4 | 5 |
| Stress at home | 0 | 1 | 2 | 3 | 4 | 5 |
| Certain intensive odors | 0 | 1 | 2 | 3 | 4 | 5 |
| Feeling tense | 0 | 1 | 2 | 3 | 4 | 5 |
| Climbing flights of stairs | 0 | 1 | 2 | 3 | 4 | 5 |
| Depressed mood | 0 | 1 | 2 | 3 | 4 | 5 |
| Smell of paint | 0 | 1 | 2 | 3 | 4 | 5 |
| Sport activities | 0 | 1 | 2 | 3 | 4 | 5 |
| Perfumes | 0 | 1 | 2 | 3 | 4 | 5 |
| Arguments with people | 0 | 1 | 2 | 3 | 4 | 5 |
| Having the flu | 0 | 1 | 2 | 3 | 4 | 5 |
| Sinus problems | 0 | 1 | 2 | 3 | 4 | 5 |
| Being excited | 0 | 1 | 2 | 3 | 4 | 5 |
| Intense worries | 0 | 1 | 2 | 3 | 4 | 5 |
| Feeling unhappy | 0 | 1 | 2 | 3 | 4 | 5 |
| Dogs | 0 | 1 | 2 | 3 | 4 | 5 |
| Overexertion | 0 | 1 | 2 | 3 | 4 | 5 |
| Viruses | 0 | 1 | 2 | 3 | 4 | 5 |
| Feeling weak | 0 | 1 | 2 | 3 | 4 | 5 |
| Feathers | 0 | 1 | 2 | 3 | 4 | 5 |
| Cats | 0 | 1 | 2 | 3 | 4 | 5 |
| House dust | 0 | 1 | 2 | 3 | 4 | 5 |

39. Do you identify your asthma triggers through symptoms such as shortness of breath, chest pain, wheezing, and/or coughing?

- Yes
- No
- I’m not sure

40. Do you do anything to try avoid your asthma triggers?

- Yes
- No

Branching Logic: If ‘Yes’ selected: What do you do to avoid your triggers?

SELECT ALL THAT APPLY

[ ] Use an inhaler

[ ] Avoiding smoking or avoid others who are smoking

[ ] Ventilate your home/ place or work by opening windows or use of an air purifier

[ ] Take allergy medication

[ ] Avoid physical activity

[ ] Stay inside during days with poor air quality

[ ] Nothing

[ ] Other, Specify _____________________________________________

41. How do you talk with healthcare providers about triggers for your asthma?

[ ] At my annual health visits with my primary care provider

[ ] Over the phone or through my patient portal

[ ] During emergency room visits

[ ] I don’t discuss my asthma triggers with healthcare providers

42. Has a health professional ever advised you to change things in your home, school, or work to improve your asthma?
    - Yes
    - No
    - I’m not sure

Branching Logic: If ‘Yes’ selected: What information or guidance, if any, have healthcare professionals offered to help manage your asthma triggers?

SELECT ALL THAT APPLY

[ ] Use and refill my inhaler prescription regularly

[ ] Avoid smoking or those who smoke

[ ] Ventilate my home or place of work

[ ] Clean heat and air conditioning vents regularly

[ ] Stay inside during days with poor air quality

[ ] Maintain mold control

[ ] Other, Specify _____________________________________________

43. If you could give someone advice on how to avoid their asthma triggers, what would it be?

Text box: _____________________________________________

**Survey Termination**

[Name], we thank you for completing this survey! We appreciate that you contributed your valuable time and your honest information to help us better understand how the environment affects people with asthma.

Within one business day, you will receive an email to claim a $30 virtual visa gift card incentive. This email will come from Clincard, which is the payment system that the University of Pennsylvania uses to pay research participants. After clicking on the link to access your Clincard, you will be asked to enter a 4-digit access code. This access code is your year of birth.
