# Supplementary material for: Clustering of self-reported social and environmental risk factors identifies vulnerable subgroups with increased asthma morbidity
Source: J Allergy Clin Immunol Glob. 2026 Aug 14;5(6):100775. doi: 10.1016/j.jacig.2026.100775 (PMC13545429; doi:10.1016/j.jacig.2026.100775)
Supplement: Supplementary file 6 [file mmc6.docx]

**S3 Text. Supplemental Results**

**Survey administration**

A diagram showing the number of people at each stage of the survey procedure is provided in Figure S1. Of 17,110 Penn Medicine health system patients deemed eligible for participation in the study, 6,512 were invited to participate in 18 total rounds of survey deployment, resulting in 974 responses from participants meeting all inclusion and exclusion criteria (response rate 15.0%). Bivariate analyses comparing the distribution of demographic characteristics for study participants versus survey nonrespondents found that recruitment rate and survey completion was higher in respondents who were female (72% vs 63%) and of White race (42% vs 32%) (Table S1). The distribution of study participants’ sex and race/ethnicity reflected that of the eligible Penn Medicine adults with asthma, with a purposeful oversampling of minority race groups.

**Multicollinearity of demographic, trigger subscale score, and SDOH variables**

No strong (r < 0.70) correlations were observed among independent variables, but several moderate positive correlations (0.50 < r < 0.70) were present between pairs of trigger subscale scores (Figure S2). Weak positive correlations (0 < r < 0.40) were observed between many of the SDOH variables, as well as between SDOH variables and trigger subscale scores, with the strongest among them observed between food insecurity and transportation insecurity (r = 0.46) and between food insecurity and Medicaid insurance (r = 0.43).

**Selection of age variable levels for regression models**

The distributions of the log count of ED visits versus age for multivariable ZIP models fit with natural cubic splines of age containing 3-5 knots are shown in Figure S3. Based on these distributions, we re-leveled age into three categories (18-44, 45-64, and 65+ years) for all regression analyses.
