# Supplementary material for: Clustering of self-reported social and environmental risk factors identifies vulnerable subgroups with increased asthma morbidity
Source: J Allergy Clin Immunol Glob. 2026 Aug 14;5(6):100775. doi: 10.1016/j.jacig.2026.100775 (PMC13545429; doi:10.1016/j.jacig.2026.100775)
Supplement: Supplementary file 7 [file mmc7.docx]

**ONLINE REPOSITORY**

**Clustering of Self-Reported Social and Environmental Risk Factors Identifies Vulnerable Subgroups with Increased Asthma Morbidity**

Alana Schreibman^1^, Kimberly Lactaoen^2^, Anuradha L. Vyas,^2^ Lizbeth F. Gómez^2^, Aurora Mills^2^, Patrick K. Gleeson^3^, Andrea J. Apter^3^, Rebecca A. Hubbard^4^, Gary E. Weissman^2,3^, Blanca E. Himes^5^

**Supplemental Figure Legends**

**Figure S1. Study flow diagram.** Shown are the numbers of people at each stage of the survey procedure, from recruitment to study inclusion. *SABA*, short-acting β_2_ agonist; *ICD-10*, International Classification of Diseases, 10^th^ Revision; *EHR*, electronic health record.

**Figure S2. Pairwise correlation between all variables included in zero-inflated negative binomial regression models.** Measures in each box correspond to Pearson’s correlation coefficients. For nominal categorical variables, reference levels were set as follows: White (race and ethnicity), none (smoking history), not overweight or obese (BMI), no (COPD), private (health insurance type), high school or below (education), no (housing insecurity, food insecurity, transportation insecurity, energy insecurity, phone insecurity, violence). Unknown/Other levels are not shown. *COPD*, chronic obstructive pulmonary disease; *BMI*, body mass index.

**Figure S3. Spline analysis for age in zero-inflated Poisson regression models.** Shown are the log counts of asthma-related emergency department (ED) visits for multivariable zero-inflated Poisson regression models plotted against splines of age with varying knots. All other terms were held constant at their reference level.

**Table S1. Characteristics of study participants versus survey nonrespondents and all eligible participants.**

Shown are the number and percentage of people in each level for categorical variables, and the median and interquartile range (IQR) for discrete variables for all eligible participants, study participants, and survey nonrespondents. Surveys were deployed in batches between April 2023 and May 2024, with the aim of recruiting a cohort with demographics representative of the underlying Penn Medicine asthma patient population according to EHR variables for sex, race and ethnicity. Characteristics shown correspond to participant information in the Penn Medicine EHR used for recruitment.

| **Characteristic** | **Eligible Participants** N = 17,110 | **Study participants**  N = 974 | **Nonrespondents**  N = 5,536 | **p-value***^a^* |
| --- | --- | --- | --- | --- |
| Age | 48 (33,63) | 46 (33, 60) | 45 (32, 61) | <10⁻⁴ |
| Sex |  |  |  | <10⁻⁴ |
| Male | 5,112 (30%) | 273 (28%) | 2,060 (37%) |  |
| Female | 11,996 (70%) | 701 (72%) | 3,475 (63%) |  |
| Other | 2 (<0.1%) | 0 (0%) | 1 (<0.1%) |  |
| Race |  |  |  | <10⁻⁴ |
| American Indian or Alaskan Native | 24 (0.1%) | 6 (0.6%) | 18 (0.3%) |  |
| Asian | 484 (2.8%) | 44 (4.5%) | 284 (5.1%) |  |
| Black | 6,689 (39%) | 421 (43%) | 2,774 (50%) |  |
| Native Hawaiian or Pacific Islander | 25 (0.1%) | 4 (0.4%) | 14 (0.3%) |  |
| White | 8,203 (48%) | 410 (42%) | 1,786 (32%) |  |
| Other | 742 (4.3%) | 50 (5.1%) | 384 (6.9%) |  |
| Unknown | 943 (5.5%) | 39 (4.0%) | 276 (5.0%) |  |
| Ethnicity |  |  |  | 0.0090 |
| Not Hispanic or Latino | 15,917 (93%) | 904 (93%) | 5,085 (92%) |  |
| Hispanic or Latino | 904 (5.3%) | 53 (5.4%) | 364 (6.6%) |  |
| Unknown | 289 (1.7%) | 17 (1.7%) | 87 (1.6%) |  |

*^a^*Kruskal-Wallis rank sum test; Pearson’s Chi-squared test comparing study participants versus nonrespondents

**Table S2. Principal component analysis of asthma trigger variables.**

Shown are the loadings corresponding to each asthma trigger resulting from a five-factor principal component analysis solution with Varimax rotation. Based on the distribution of loadings between factors, each factor was found to correspond to a trigger theme: air pollution/irritant triggers (factor 1), psychological triggers (factor 2), allergen triggers (factor 3), physical activity triggers (factor 4), and infection triggers (factor 5).

|  | **Factor** | | | | |
| --- | --- | --- | --- | --- | --- |
| **Trigger** | **1** | **2** | **3** | **4** | **5** |
| Having a cold | 0.15 | 0.14 | 0.12 | 0.16 | 0.86 |
| Cigarette smoke | 0.50 | 0.04 | 0.29 | 0.15 | 0.45 |
| Running | 0.14 | 0.13 | 0.22 | 0.78 | 0.34 |
| Being angry | 0.22 | 0.70 | 0.09 | 0.24 | 0.18 |
| Pollen | 0.25 | 0.11 | 0.39 | 0.28 | 0.54 |
| Feeling alone | 0.00 | 0.77 | 0.07 | -0.06 | 0.07 |
| Exhaust fumes | 0.63 | 0.33 | 0.14 | 0.26 | 0.32 |
| Bicycle riding | 0.15 | 0.19 | 0.15 | 0.80 | 0.14 |
| Stress at home | 0.16 | 0.78 | 0.11 | 0.18 | 0.15 |
| Certain intensive odors | 0.71 | 0.22 | 0.22 | 0.20 | 0.36 |
| Feeling tense | 0.14 | 0.83 | 0.06 | 0.16 | 0.12 |
| Climbing flights of stairs | 0.32 | 0.29 | 0.09 | 0.66 | 0.25 |
| Depressed mood | 0.12 | 0.84 | 0.04 | 0.09 | 0.06 |
| Smell of paint | 0.76 | 0.30 | 0.19 | 0.19 | 0.16 |
| Sport activities | 0.14 | 0.16 | 0.18 | 0.84 | 0.26 |
| Perfumes | 0.77 | 0.19 | 0.24 | 0.12 | 0.19 |
| Arguments with people | 0.19 | 0.77 | 0.03 | 0.22 | 0.13 |
| Having the flu | 0.17 | 0.18 | 0.12 | 0.17 | 0.86 |
| Sinus problems | 0.19 | 0.22 | 0.18 | 0.24 | 0.76 |
| Being excited | 0.26 | 0.58 | 0.05 | 0.29 | 0.11 |
| Intense worries | 0.11 | 0.84 | 0.11 | 0.07 | 0.11 |
| Feeling unhappy | 0.10 | 0.84 | 0.09 | -0.01 | 0.08 |
| Dogs | 0.14 | 0.15 | 0.80 | 0.15 | 0.18 |
| Viruses | 0.24 | 0.18 | 0.15 | 0.20 | 0.82 |
| Feeling weak | 0.19 | 0.60 | 0.21 | 0.18 | 0.22 |
| Feathers from birds | 0.44 | 0.28 | 0.58 | 0.12 | 0.07 |
| Cats | 0.21 | 0.05 | 0.83 | 0.16 | 0.22 |
| House dust | 0.30 | 0.14 | 0.58 | 0.26 | 0.44 |

**Table S3. Characteristics of complete cases and participants excluded due to missingness.**

Shown are the number and percentage of participants in each level for categorical variables, the mean and standard deviation (SD) for continuous variables, and the median and interquartile range (IQR) for discrete variables for complete cases versus those excluded due to missingness. Although sample size varied between analyses depending on the variables included, n = 839 corresponds to the maximum possible number of participants when excluding others due to missingness in the ED visit outcome variable and the following independent variables: all five trigger subscales, smoking history, education, food insecurity, transportation insecurity.

|  | **Complete Case** | |  |
| --- | --- | --- | --- |
| **Characteristic** | **Yes**  N = 839 | **No**  N = 135 | **p-value***^a^* |
| **Demographics & Comorbidities** | | | |
| Age (years) | 46 (33, 60) | 57 (40, 67) | <10⁻⁴ |
| Sex |  |  | 0.89 |
| Male | 234 (28%) | 39 (29%) |  |
| Female | 605 (72%) | 96 (71%) |  |
| Race and ethnicity |  |  | 0.12 |
| American Indian or Alaskan Native | 5 (0.6%) | 1 (0.7%) |  |
| Asian | 46 (5.5%) | 5 (3.7%) |  |
| Black | 325 (39%) | 68 (50%) |  |
| Hispanic or Latino | 31 (3.7%) | 2 (1.5%) |  |
| White | 346 (41%) | 53 (39%) |  |
| Middle Eastern or North African | 6 (0.7%) | 0 (0%) |  |
| Native Hawaiian or Pacific Islander | 0 (0%) | 0 (0%) |  |
| Multiracial | 65 (7.7%) | 6 (4.4%) |  |
| Unknown | 15 (1.8%) | 0 (0%) |  |
| Smoking history |  |  | <10⁻⁴ |
| None | 541 (64%) | 71 (53%) |  |
| Current | 47 (5.6%) | 13 (9.6%) |  |
| Past | 204 (24%) | 34 (25%) |  |
| Passive | 47 (5.6%) | 5 (3.7%) |  |
| Unknown | 0 (0%) | 12 (8.9%) |  |
| BMI |  |  | 0.65 |
| Not overweight or obese | 211 (25%) | 28 (21%) |  |
| Overweight | 236 (28%) | 41 (30%) |  |
| Class 1 obesity | 160 (19%) | 28 (21%) |  |
| Class 2 obesity | 108 (13%) | 13 (9.6%) |  |
| Class 3 obesity | 109 (13%) | 22 (16%) |  |
| Unknown | 15 (1.8%) | 3 (2.2%) |  |
| COPD |  |  | 0.070 |
| No | 720 (86%) | 108 (80%) |  |
| Yes | 82 (9.8%) | 15 (11%) |  |
| Unknown | 37 (4.4%) | 12 (8.9%) |  |
| **Trigger Subscale Scores** | | | |
| Psychological | 0.30 (0.00, 1.00) | 0.94 (0.00, 2.00) | 0.017 |
| Unknown | 0 | 73 |  |
| Air pollution/Irritants | 1.60 (0.60, 2.25) | 2.20 (1.00, 3.00) | <10⁻⁴ |
| Unknown | 0 | 52 |  |
| Physical activity | 1.75 (0.75, 2.50) | 2.00 (1.25, 3.00) | 0.0092 |
| Unknown | 0 | 45 |  |
| Allergens | 1.80 (0.80, 2.60) | 2.37 (1.45, 3.00) | <10⁻⁴ |
| Unknown | 0 | 31 |  |
| Infection | 2.25 (1.33, 3.25) | 2.42 (2.00, 3.00) | 0.22 |
| Unknown | 0 | 29 |  |
| **Social Determinants of Health** | | | |
| Health insurance type |  |  | 0.41 |
| Private | 513 (61%) | 72 (53%) |  |
| Medicaid | 190 (23%) | 37 (27%) |  |
| Medicare | 96 (11%) | 19 (14%) |  |
| Military | 4 (0.5%) | 1 (0.7%) |  |
| None | 13 (1.5%) | 1 (0.7%) |  |
| Other | 6 (0.7%) | 0 (0%) |  |
| Unknown | 17 (2.0%) | 5 (3.7%) |  |
| Education |  |  | <10⁻⁴ |
| High school or below | 166 (20%) | 32 (24%) |  |
| Some college | 204 (24%) | 38 (28%) |  |
| Bachelor | 236 (28%) | 22 (16%) |  |
| Advanced degree | 233 (28%) | 35 (26%) |  |
| Unknown | 0 (0%) | 8 (5.9%) |  |
| Housing insecurity |  |  | 0.089 |
| No | 791 (94%) | 123 (91%) |  |
| Yes | 31 (3.7%) | 5 (3.7%) |  |
| Unknown | 17 (2.0%) | 7 (5.2%) |  |
| Food insecurity |  |  | 0.0019 |
| No | 579 (69%) | 93 (69%) |  |
| Yes | 260 (31%) | 40 (30%) |  |
| Unknown | 0 (0%) | 2 (1.5%) |  |
| Transportation insecurity |  |  | <10⁻⁴ |
| No | 719 (86%) | 115 (85%) |  |
| Yes | 120 (14%) | 14 (10%) |  |
| Unknown | 0 (0%) | 6 (4.4%) |  |
| Energy insecurity |  |  | 0.37 |
| No | 783 (93%) | 130 (96%) |  |
| Yes | 50 (6.0%) | 4 (3.0%) |  |
| Unknown | 6 (0.7%) | 1 (0.7%) |  |
| Communication insecurity |  |  | 0.90 |
| No | 767 (91%) | 125 (93%) |  |
| Yes | 65 (7.7%) | 9 (6.7%) |  |
| Unknown | 7 (0.8%) | 1 (0.7%) |  |
| Exposure to violence |  |  | 0.0019 |
| No | 681 (81%) | 112 (83%) |  |
| Yes | 153 (18%) | 18 (13%) |  |
| Unknown | 5 (0.6%) | 5 (3.7%) |  |

*^a^*Kruskal-Wallis rank sum test; Pearson’s Chi-squared test

**Table S4. Associations between individual trigger subscales and emergency department visits before and after adjusting for social determinants of health variables.**

Shown are the adjusted incidence rate ratios (IRRs), 95% confidence intervals (CIs), and p-values for zero-inflated Poisson (ZIP) regression models of emergency department visits as a count outcome. Each row corresponds to a ZIP model adjusted for one of the five trigger subscale scores with age, sex, race and ethnicity, and body mass index as covariates. All eight social determinants of health (SDOH) variables were then added to each model as additional covariates.

|  | SDOH-Unadjusted | | | | SDOH-Adjusted | | | |
| --- | --- | --- | --- | --- | --- | --- | --- | --- |
| **Trigger Subscale** | **N** | **IRR** | **95% CI** | **p-value** | **N** | **IRR** | **95% CI** | **p-value** |
| Psychological | 863 | 1.41 | 1.29, 1.55 | <10⁻⁴ | 863 | 1.24 | 1.11, 1.39 | 1.5x10⁻⁴ |
| Air pollution/Irritants | 882 | 1.28 | 1.17, 1.40 | <10⁻⁴ | 882 | 1.14 | 1.04, 1.26 | 0.0063 |
| Physical activity | 892 | 1.28 | 1.17, 1.41 | <10⁻⁴ | 892 | 1.11 | 1.00, 1.22 | 0.051 |
| Allergens | 903 | 1.25 | 1.14, 1.38 | <10⁻⁴ | 903 | 1.19 | 1.07, 1.31 | 7.9x10⁻⁴ |
| Infection | 905 | 1.42 | 1.28, 1.58 | <10⁻⁴ | 905 | 1.27 | 1.15, 1.40 | <10⁻⁴ |

**Table S5. SDOH associated with individual trigger subscale scores in linear regression models.**

Shown are the adjusted odds ratios (ORs), 95% confidence intervals (CIs), and p-values for linear regression models of each trigger subscale score as a continuous outcome. Each group of columns corresponds to one linear regression model, with one of the five trigger subscales as the dependent variable, all eight SDOH variables as independent variables, and age, sex, and BMI as covariates.

|  | **Psychological**  **(N = 901)** | | **Air pollution/Irritants**  **(N = 922)** | | **Physical activity**  **(N = 929)** | | **Allergens**  **(N = 943)** | | **Infection**  **(N = 945)** | |
| --- | --- | --- | --- | --- | --- | --- | --- | --- | --- | --- |
| **SDOH** | **Beta [95% CI]** | **p-value** | **Beta [95% CI]** | **p-value** | **Beta [95% CI]** | **p-value** | **Beta [95% CI]** | **p-value** | **Beta [95% CI]** | **p-value** |
| Health insurance type |  |  |  |  |  |  |  |  |  |  |
| Private | — |  | — |  | — |  | — |  | — |  |
| Medicaid | 0.33 [0.17, 0.49] | <10⁻⁴ | 0.17 [-0.03, 0.38] | 0.095 | 0.11 [-0.10, 0.32] | 0.29 | 0.19 [-0.02, 0.40] | 0.074 | 0.28 [0.06, 0.51] | 0.013 |
| Medicare | 0.08 [-0.11, 0.28] | 0.39 | -0.07 [-0.32, 0.17] | 0.57 | -0.20 [-0.45, 0.06] | 0.13 | 0.02 [-0.23, 0.27] | 0.86 | 0.13 [-0.14, 0.39] | 0.35 |
| Unknown/Other | -0.10 [-0.36, 0.16] | 0.44 | -0.10 [-0.43, 0.24] | 0.58 | -0.35 [-0.70, 0.00] | 0.051 | 0.01 [-0.33, 0.35] | 0.95 | -0.14 [-0.50, 0.22] | 0.45 |
| Education |  |  |  |  |  |  |  |  |  |  |
| High school or below | — |  | — |  | — |  | — |  | — |  |
| Some college | -0.14 [-0.31, 0.02] | 0.087 | -0.05 [-0.26, 0.16] | 0.64 | -0.07 [-0.29, 0.15] | 0.52 | -0.08 [-0.29, 0.13] | 0.47 | -0.10 [-0.33, 0.13] | 0.40 |
| Bachelor | -0.20 [-0.38, -0.02] | 0.027 | -0.16 [-0.38, 0.07] | 0.18 | -0.14 [-0.37, 0.10] | 0.25 | -0.07 [-0.30, 0.16] | 0.55 | -0.06 [-0.31, 0.18] | 0.62 |
| Advanced degree | -0.28 [-0.46, -0.10] | 0.0023 | -0.17 [-0.41, 0.06] | 0.14 | -0.27 [-0.51, -0.03] | 0.028 | -0.08 [-0.32, 0.16] | 0.50 | -0.02 [-0.27, 0.23] | 0.87 |
| Unknown | -0.54 [-1.2, 0.09] | 0.095 | -0.30 [-1.1, 0.52] | 0.47 | -0.81 [-1.7, 0.04] | 0.061 | -0.50 [-1.3, 0.35] | 0.25 | -0.65 [-1.6, 0.25] | 0.15 |
| Housing insecurity |  |  |  |  |  |  |  |  |  |  |
| No | — |  | — |  | — |  | — |  | — |  |
| Yes | 0.26 [-0.05, 0.56] | 0.100 | -0.04 [-0.43, 0.35] | 0.84 | -0.06 [-0.48, 0.36] | 0.78 | -0.08 [-0.48, 0.33] | 0.71 | 0.03 [-0.41, 0.46] | 0.90 |
| Unknown | -0.39 [-0.76, -0.02] | 0.039 | -0.41 [-0.88, 0.05] | 0.083 | -0.42 [-0.92, 0.08] | 0.098 | -0.39 [-0.87, 0.10] | 0.12 | -0.22 [-0.72, 0.28] | 0.39 |
| Food insecurity |  |  |  |  |  |  |  |  |  |  |
| No | — |  | — |  | — |  | — |  | — |  |
| Yes | 0.18 [0.03, 0.33] | 0.018 | 0.39 [0.20, 0.58] | <10⁻⁴ | 0.30 [0.10, 0.49] | 0.0031 | 0.19 [-0.01, 0.38] | 0.058 | 0.19 [-0.02, 0.39] | 0.077 |
| Unknown | -0.91 [-2.5, 0.71] | 0.27 | 1.0 [-1.1, 3.1] | 0.34 | -1.5 [-3.6, 0.71] | 0.19 | 0.88 [-1.3, 3.0] | 0.43 | -0.45 [-2.8, 1.9] | 0.70 |
| Transportation insecurity |  |  |  |  |  |  |  |  |  |  |
| No | — |  | — |  | — |  | — |  | — |  |
| Yes | 0.25 [0.06, 0.44] | 0.0087 | 0.11 [-0.13, 0.35] | 0.37 | 0.12 [-0.13, 0.36] | 0.36 | 0.18 [-0.07, 0.43] | 0.15 | 0.12 [-0.14, 0.39] | 0.36 |
| Unknown | 0.01 [-0.72, 0.74] | 0.98 | -0.71 [-1.7, 0.23] | 0.14 | 0.10 [-0.88, 1.1] | 0.84 | -0.67 [-1.6, 0.30] | 0.18 | -0.27 [-1.3, 0.76] | 0.60 |
| Energy insecurity |  |  |  |  |  |  |  |  |  |  |
| No | — |  | — |  | — |  | — |  | — |  |
| Yes | 0.18 [-0.08, 0.43] | 0.18 | 0.07 [-0.27, 0.41] | 0.68 | 0.05 [-0.30, 0.39] | 0.79 | 0.00 [-0.34, 0.34] | 1.00 | 0.21 [-0.15, 0.58] | 0.25 |
| Unknown | -0.02 [-0.69, 0.64] | 0.95 | -0.39 [-1.3, 0.46] | 0.37 | 0.55 [-0.34, 1.4] | 0.23 | -0.40 [-1.3, 0.49] | 0.38 | -0.05 [-0.99, 0.90] | 0.93 |
| Communication insecurity |  |  |  |  |  |  |  |  |  |  |
| No | — |  | — |  | — |  | — |  | — |  |
| Yes | 0.01 [-0.23, 0.24] | 0.95 | -0.17 [-0.48, 0.13] | 0.26 | 0.03 [-0.29, 0.35] | 0.86 | 0.08 [-0.23, 0.39] | 0.61 | -0.01 [-0.34, 0.33] | 0.97 |
| Unknown | -0.22 [-0.84, 0.40] | 0.48 | -0.52 [-1.3, 0.27] | 0.20 | -0.65 [-1.5, 0.18] | 0.12 | -0.67 [-1.5, 0.16] | 0.11 | -0.51 [-1.4, 0.36] | 0.25 |
| Exposure to violence |  |  |  |  |  |  |  |  |  |  |
| No | — |  | — |  | — |  | — |  | — |  |
| Yes | 0.13 [-0.02, 0.28] | 0.078 | 0.15 [-0.04, 0.34] | 0.11 | 0.19 [-0.01, 0.38] | 0.063 | 0.19 [-0.01, 0.38] | 0.062 | 0.27 [0.06, 0.48] | 0.011 |
| Unknown | 0.09 [-0.50, 0.67] | 0.77 | -0.06 [-0.82, 0.70] | 0.88 | -0.11 [-0.89, 0.68] | 0.79 | 0.40 [-0.34, 1.1] | 0.29 | -0.19 [-1.0, 0.64] | 0.66 |

**Table S6. Participant responses to care-related behavior questions by cluster.**

Shown are the number and percentage of study participants in each level of all categorical variables across clusters. Characteristics shown correspond to variables derived from participants’ survey responses but not used as inputs in hierarchical clustering, which include care-related behaviors such as methods of seeking asthma care, discussing asthma triggers with healthcare teams, and obtaining asthma medications. “Method of discussing triggers with healthcare team” and “method of obtaining asthma medications” were individual survey questions which allowed participants to select multiple answers; responses were therefore coded as indicator variables reflecting the percentage of participants who selected each option and may sum to more than 100%. *ED*, emergency department.

|  |  | **Cluster** | | | |  |
| --- | --- | --- | --- | --- | --- | --- |
| **Characteristic** | **Overall**  N = 974 | **1**  N = 73 | **2**  N = 224 | **3**  N = 227 | **4**  N = 450 | **p-value***^a^* |
| **Method of Seeking Asthma Care** | | | | | | |
| Location |  |  |  |  |  | <10⁻⁴ |
| Primary care office | 464 (48%) | 23 (32%) | 84 (38%) | 99 (44%) | 258 (57%) |  |
| Specialist office | 74 (7.6%) | 0 (0%) | 6 (2.7%) | 10 (4.4%) | 58 (13%) |  |
| Urgent care | 109 (11%) | 8 (11%) | 19 (8.5%) | 29 (13%) | 53 (12%) |  |
| Emergency room | 274 (28%) | 38 (52%) | 108 (48%) | 78 (34%) | 50 (11%) |  |
| Unknown/Other | 53 (5.4%) | 4 (5.5%) | 7 (3.1%) | 11 (4.8%) | 31 (6.9%) |  |
| **Method of Discussing Triggers with Healthcare Team** | | | | | | |
| Annual visits | 766 (79%) | 46 (63%) | 173 (77%) | 195 (86%) | 352 (78%) | 4.3x10⁻⁴ |
| Phone | 286 (29%) | 16 (22%) | 97 (43%) | 73 (32%) | 100 (22%) | <10⁻⁴ |
| Emergency room visits | 168 (17%) | 24 (33%) | 73 (33%) | 38 (17%) | 33 (7.3%) | <10⁻⁴ |
| Never | 94 (9.7%) | 6 (8.2%) | 12 (5.4%) | 15 (6.6%) | 61 (14%) | 0.0017 |
| **Method of Obtaining Asthma Medications** | | | | | | |
| Doctor prescription | 892 (92%) | 64 (88%) | 200 (89%) | 213 (94%) | 415 (92%) | 0.19 |
| ED prescription | 76 (7.8%) | 14 (19%) | 40 (18%) | 13 (5.7%) | 9 (2.0%) | <10⁻⁴ |
| Doctor sample | 14 (1.4%) | 1 (1.4%) | 7 (3.1%) | 2 (0.9%) | 4 (0.9%) | 0.11 |
| ED sample | 9 (0.9%) | 2 (2.7%) | 6 (2.7%) | 1 (0.4%) | 0 (0%) | 0.0019 |
| Borrowing | 39 (4.0%) | 6 (8.2%) | 20 (8.9%) | 11 (4.8%) | 2 (0.4%) | <10⁻⁴ |
| Other | 37 (3.8%) | 1 (1.4%) | 9 (4.0%) | 7 (3.1%) | 20 (4.4%) | 0.56 |

*^a^*Kruskal-Wallis rank sum test; Pearson’s Chi-squared test
